# Supplementary material for: Higher-order singularities in phase-tracked electromechanical oscillators
Source: Nat Commun. 2023 Dec 1;14:7944. doi: 10.1038/s41467-023-43708-y (PMC10692225; doi:10.1038/s41467-023-43708-y)
Supplement: Supplementary file 1 — Supplementary Information [file 41467_2023_43708_MOESM1_ESM.pdf]

Higher-order singularities in phase-tracked electromechanical oscillators

Xin Zhou et al.

(Dated: November 15, 2023)

CONTENTS

SUPPLEMENTARY NOTE 1. Experiment details 3
A. Device description 3
B. Experimental setup 4

SUPPLEMENTARY NOTE 2. Coriolis coupling 7

SUPPLEMENTARY NOTE 3. Phase-tracked closed-loop oscillation 12
A. Equations of motion and open-loop responses 12
B. Phase-tracked oscillation 14
C. “Pitchfork” bifurcation and singularity nexus 15

SUPPLEMENTARY NOTE 4. Stability analysis 17

SUPPLEMENTARY NOTE 5. Cubic singularity 19

SUPPLEMENTARY NOTE 6. Classical Bloch sphere 20

SUPPLEMENTARY NOTE 7. Order parameter 22

SUPPLEMENTARY NOTE 8. High sensitivity at singularity nexus 22
A. Sensitivity at singularity nexus 22
B. Sensitivity comparison with binary exceptional point and diabolic point 24

SUPPLEMENTARY DISCUSSION 1. Voltage-controlled nonreciprocal state transfer 26
A. Concept of the nonreciprocal state transfer 26
B. Experimental demonstration of the nonreciprocal state transfer 28

SUPPLEMENTARY DISCUSSION 2. Phase-tracked cubic singularities in linear coherent coupling 31

SUPPLEMENTARY REFERENCES. 36

SUPPLEMENTARY NOTE 1. EXPERIMENT DETAILS

A. Device description

A microelectromechanical silicon disk resonator with eight-fold symmetry is used as the experimental testbed [1]. The device is the core of an established high-performance MEMS gyroscope called the disk resonator gyroscope [1–5]. Owing to its excellent integration, superb controllability&tuneability, good stability, and high signal-to-noise ratio of displacement measurement, the micro disk resonator can be a very convenient experimental platform for basic research [6, 7]. The micrograph of one of the devices is shown in Supplementary Fig. **1**a. The device used in the experiment is identical to that in this picture. The 3D model of the disk resonator is shown in Supplementary Fig. **1**b. The disk resonator is made up of nine concentric nested rings interconnected by radial spokes and anchored in the centre. Some movable mass blocks are attached to the four outer layers of rings and spokes. The section view of the resonator is shown in Supplementary Fig. **1**c. The deformable parts of the resonator body (light blue) are separated from the substrate by a gap. The central part of the resonator is fixed to the substrate using a bonding process. The resonator is released from the blocks that are connected to the substrate using a deep reactive ion etching process. Sixteen fixed blocks around the resonator are used as capacitive electrodes to actuate, transduce, and tune the disk resonator. A more detailed description of the design of this disk resonator can be found in ref. [1]. The external electric signals are applied by wire bonding to the metal pads. The resonator is capsulated in a ceramic leaded chip carrier. The medium-vacuum environment in the sealed package (about 2 Pa) can produce an appropriate air damping for this study.

Based on the linear theory of elasticity, the vibrating resonator body has a set of eigenstates, which refer to the displacement patterns of the normal modes. Here, a pair of near-degenerate four-node standing-wave modes labelled by modes 1 and 2 with natural frequencies $\omega_{1,2}/2\pi \approx 3.85$ kHz are considered (Supplementary Fig. **2**). The deformations of the modes are perfectly in-plane, which thereby can be actuated and transduced by lateral capacitive electrodes. Each standing-wave mode can be regarded as a combination of two in-plane whispering gallery travelling-wave modes that circularly propagate in opposite directions [8]. The wave number of those travelling-wave modes is equal to two.

The decaying measurements of the degenerate four-node modes are shown in Supplementary Fig. **1**d. First, we drive each mode independently to maintain a steady vibration, and then, we turn off the drive and record the free evolving signal. The decaying signals are fitted with the function $q_{\max}e^{-t/\tau}$, where $q_{\max}$ is the maximum amplitude, t is time, and τ is the decay time constant. An identical $\tau \approx 5.7$ s of the two modes is measured. The damping rate of the modes is measured to be $\gamma = 2/\tau \approx 2\pi \times 55.8$ mHz.

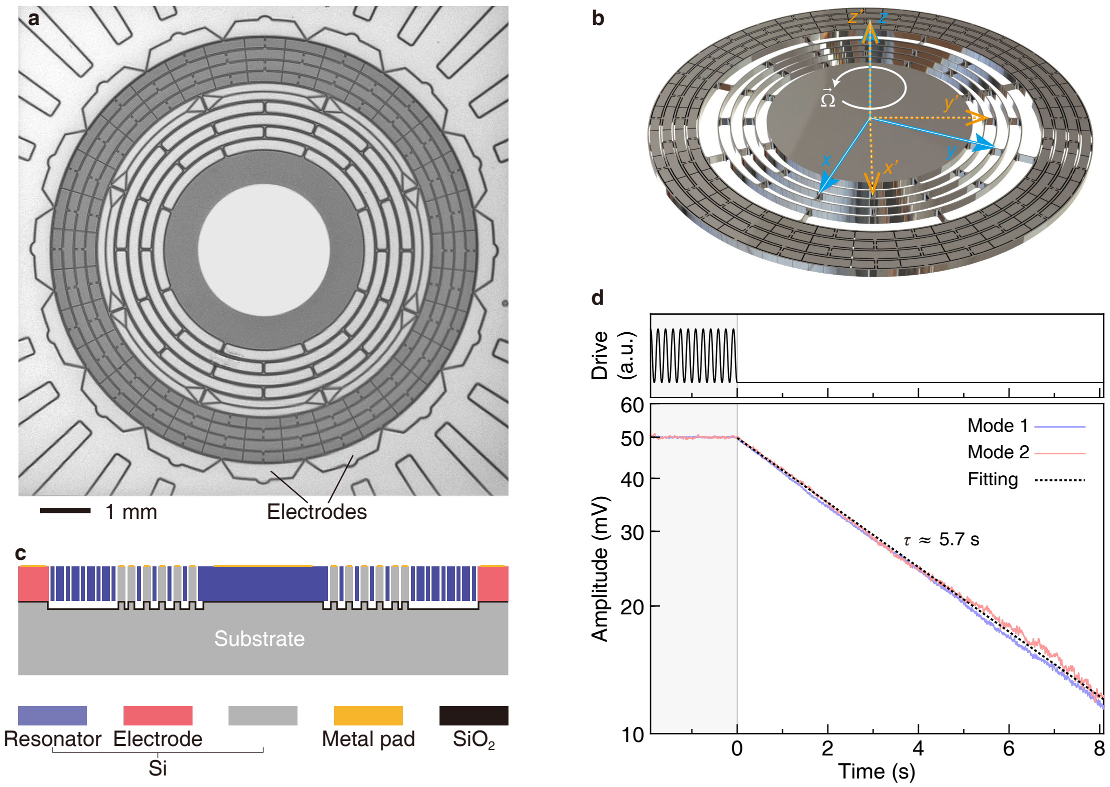

Supplementary Figure 1. **Microelectromechanical disk resonator.** (**a**) Top-view micrograph. (**b**) Three-dimensional model. (**c**) Section view. (**d**) Decaying signals of the degenerate modes show an identical damping rate.

B. Experimental setup

The experimental setup is shown in Supplementary Fig. **3**a. The microelectromechanical disk resonator can be actuated, transduced, and tuned electrostatically by the integrated capacitive electrodes. To actuate mode 1 into linear vibration, two differential alternating actuation signals generated by a lock-in amplifier (Zurich Instruments HF2LI) are applied to the antinodal electrodes of mode 1. The amplitude of the driving force is kept constant. The differential driving configuration largely eliminates the crosstalk to mode 2. A constant DC voltage $V_0 \approx 2.5$ V is applied on the resonator body to provide a reference bias for the read-out circuit. A high-frequency (2 MHz) square-wave carrier signal is applied on the resonator body to modulate the modal signals and convert them outside of the $1/f$ noise band of the electronics. The antinodal displacements of the two modes are picked up capacitively using charge amplifiers. The transduced signals are mixed with the high-frequency carrier wave and filtered by a low-pass filter (LPF) to obtain the down-converted modal signals. The two antinodal displacement signals vibrating in the quadrature of each mode are differentiated to avoid common-mode errors. The signal processing is conducted by an analogue circuit,

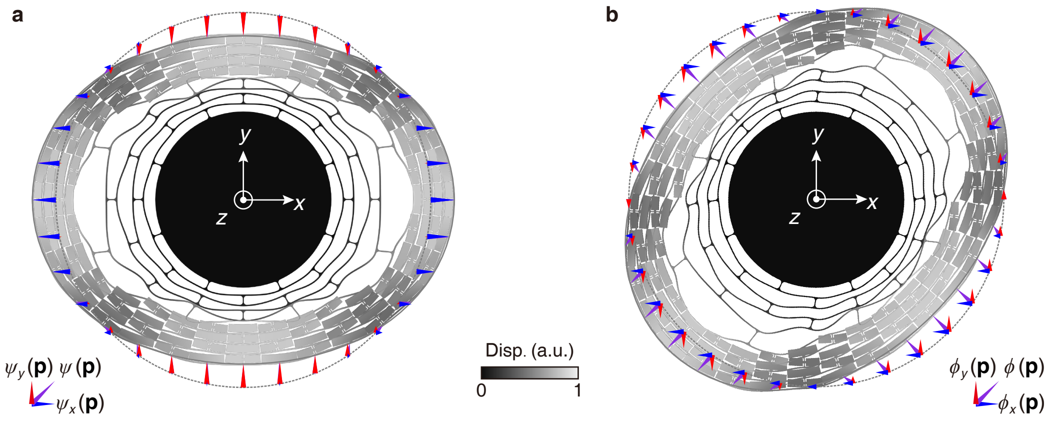

Supplementary Figure 2. **Mode shapes and distributions of the displacement.** Outline distributions of the displacement showing the normalized shape function of $\boldsymbol{\psi}$ for mode 1 (**a**) and that of $\boldsymbol{\phi}$ for mode 2 (**b**).

the picture of which is shown in Supplementary Fig. **3**b. The displacement signals $q_{1,2} = |q_{1,2}|\cos(\omega_{\rm d}t + \theta_{1,2})$ are measured by the lock-in amplifier using the Homodyne method (see methods).

The open-loop test is implemented using the parametric sweeper block of the lock-in amplifier to obtain the amplitude-frequency responses $|q_{1,2}|$ and phase-frequency responses $\theta_{1,2}$. The phase-tracked closed-loop oscillation can be realized by enabling the PLL block of the lock-in amplifier to track the driven-mode phase in quadrature $\theta_1 = -\pi/2$, in this case, the phase-tracked frequency $\omega_{\rm d}^*$, the amplitudes of both modes $|q_{1,2}|$ and the relative phase $\vartheta \equiv \theta_2 - \theta_1$ are recorded.

The programmable direct current (DC) source (IT6402) applies a DC tuning voltage $V_{\rm t}$ to the antinodal electrodes of mode 2 to introduce electrostatic negative stiffness and eventually adjust the degeneracy condition $\Delta\omega \equiv \omega_2 - \omega_1$ (see method).

The device is connected to the signal processing circuit using a package holder, as shown in Supplementary Fig. **3**b, in which an unsealed device is demonstrated (The measured device is sealed). As shown in Supplementary Fig. **3**c, the device and the circuitry are fixed to a rate table, which can produce an out-of-plane rotation with programmed angular velocity in a range of $0°/\text{s}$ to $\pm 1200°/\text{s}$. The angular velocity error of this rate table under stable rotation is $< 0.001°/\text{s}$. The load of this rate table is isolated in a temperature-controlled chamber. The temperature of our system is controlled to 25°C throughout the experiment with fluctuation < 0.01°C. The circuitry on the rate table is electrically connected to the equipment outside the chamber through slip rings inside the rate table, such that the device and circuitry can perpetually rotate without affecting the electric connection with the equipment.

The complete set of the open-loop frequency responses including the amplitudes $|q_{1,2}|$ and phases $\theta_{1,2}$ as functions of the angular velocity Ω at different degeneracy conditions ($\Delta\omega = 0$, $-\gamma$, and γ) are shown

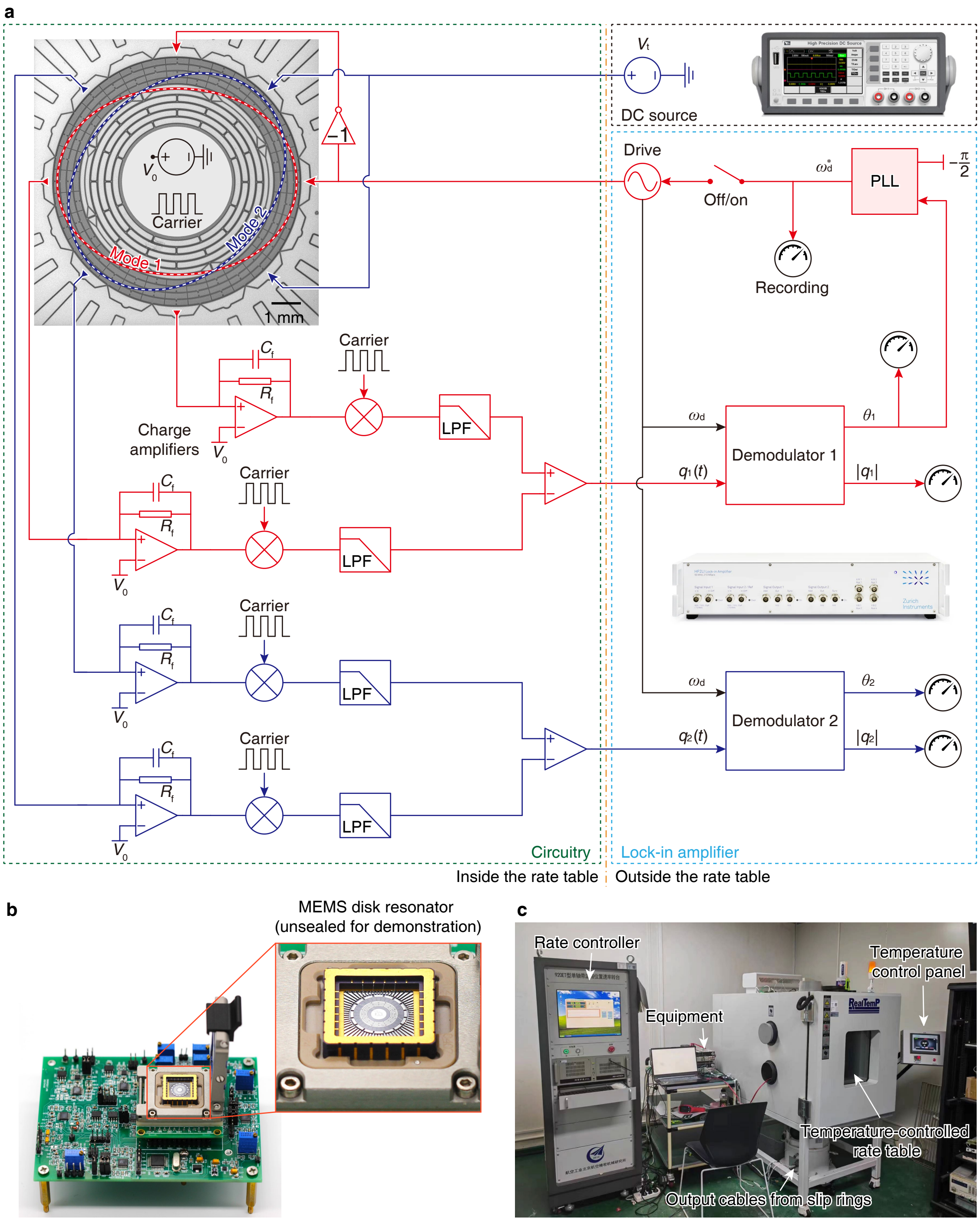

Supplementary Figure 3. **Experimental setup.** (**a**) Schematic of the experiment. The device and the signal processing circuitry are integrated into a printed circuit board. The drive, Homodyne measurement, and PLL are implemented using a lock-in amplifier. The frequency tuning signal is applied by a DC source. (**b**) Signal processing circuitry. An unsealed device is mounted on the package holder for demonstration. (**c**) Measurement environment. The device and circuitry are fixed inside the temperature-controlled rate table and are electrically connected with outside equipment through slip rings.

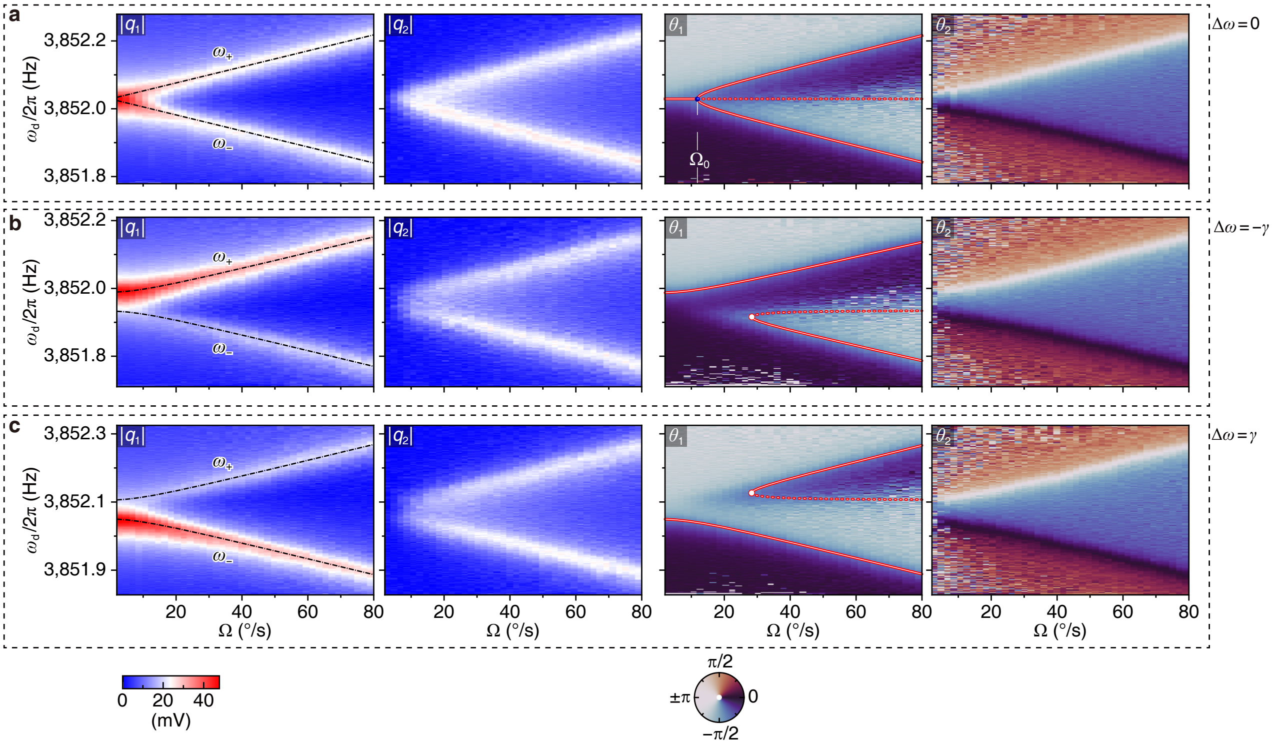

Supplementary Figure 4. **Open-loop frequency responses.** Experimental frequency responses of the amplitudes and phases versus the angular velocity Ω for the degeneracy conditions $\Delta\omega \approx 0$ (**a**), $-\gamma$ (**b**), and γ (**c**). Dot-dashed curves in the $|q_1|$ responses represent the eigenfrequencies. Red contours in the θ_1 responses indicate the theoretical $\theta_1 = -\pi/2$ closed-loop frequency ω_{d}^*, showing (a) "pitchfork" bifurcation and (b,c) saddle-node bifurcations.

in Supplementary Fig. **4**. The dot-dashed curves in the $|q_1|$ responses indicate the eigenfrequencies. The $\theta_1 = -\pi/2$ red equiphase contours in the θ_1 responses give the bifurcation patterns of the PhT frequency ω_{d}^*.

SUPPLEMENTARY NOTE 2. CORIOLIS COUPLING

The Coriolis effect introduced by the rotation will cause an interaction between modes 1 and 2. If transformed from the standing-wave to the travelling-wave basis, the Coriolis coupling is equivalent to the rotational Doppler effect [9], also regarded as an acoustic analogue of the Zeeman effect [10].

Next, we describe the theoretical model for this Coriolis coupling. We define a resonator frame x-y-z that is attached to the resonator, as shown in Supplementary Fig. **1**b. The out-of-plane rotation with an angular rate Ω of the resonator frame to the inertial reference frame x'-y'-z' is considered. The positive rotation direction is along the z axis. We consider the case where the origins of the two frames coincide, and the z and z' axes are aligned.

The displacement pattern of the vibrating mode is described by the displacement field $\mathbf{u}(\mathbf{s})$, where $\mathbf{s} = (x, y, z)^{\mathrm{T}}$ indicates the position vector of the mass points on the resonator frame. The displacement patterns of the four-node standing-wave modes are described by

$$\mathbf{u}_1(\mathbf{s}, t) = \boldsymbol{\psi}(\mathbf{s}) q_1(t), \quad \mathbf{u}_2(\mathbf{s}, t) = \boldsymbol{\phi}(\mathbf{s}) q_2(t). \tag{S.1}$$

Here, $\boldsymbol{\psi} = (\psi_x(\mathbf{s}), \psi_y(\mathbf{s}), \psi_z(\mathbf{s}))^{\mathrm{T}}$ and $\boldsymbol{\phi} = (\phi_x(\mathbf{s}), \phi_y(\mathbf{s}), \phi_z(\mathbf{s}))^{\mathrm{T}}$ indicate the normalized shape functions of the two modes, as shown in Supplementary Fig. **2**. q_1 and q_2 are the antinodal vibrating amplitudes of them. The overall displacement field of the resonator can be described by the superposition of the displacement fields of the two modes: $\mathbf{u} \equiv (u_x, u_y, u_z)^{\mathrm{T}} = \mathbf{u}_1 + \mathbf{u}_2$. The kinetic energy of the rotating and vibrating resonator is given by

$$T = \frac{1}{2} \iiint\limits_V \rho \, (\dot{\mathbf{r}} + \boldsymbol{\Omega} \times \mathbf{r})^2 \, \mathrm{d}V = \frac{1}{2} \iiint\limits_V \rho \, [\dot{\mathbf{u}} + \boldsymbol{\Omega} \times (\mathbf{s} + \mathbf{u})]^2 \, \mathrm{d}V = T_1 + T_2 + T_3, \tag{S.2}$$

where $\mathbf{r} = \mathbf{s} + \mathbf{u}$ and $\dot{\mathbf{s}} = 0$, ρ is the density of silicon, $\boldsymbol{\Omega} = (0, 0, \Omega)^{\mathrm{T}}$, and

$$T_1 = \frac{1}{2} \rho \iiint\limits_V \left(\dot{u}_x^2 + \dot{u}_y^2 + \dot{u}_z^2\right) \mathrm{d}V, \tag{S.3}$$

$$T_2 = \frac{1}{2} \rho \Omega^2 \iiint\limits_V \left[(x + u_x)^2 + \left(y + u_y\right)^2\right] \mathrm{d}V, \tag{S.4}$$

$$T_3 = \rho \Omega \iiint\limits_V \left[(x + u_x) \, \dot{u}_y - \left(y + u_y\right) \dot{u}_x\right] \mathrm{d}V. \tag{S.5}$$

Expanding T_1 we have

$$\begin{aligned} T_1 = {} & \frac{1}{2} \dot{q}_1^2 \rho \iiint\limits_V \left(\psi_x^2 + \psi_y^2 + \psi_z^2\right) \mathrm{d}V + \frac{1}{2} \dot{q}_2^2 \rho \iiint\limits_V \left(\phi_x^2 + \phi_y^2 + \phi_z^2\right) \mathrm{d}V \\ & + \dot{q}_1 \dot{q}_2 \rho \iiint\limits_V \left(\psi_x \phi_x + \psi_y \phi_y + \psi_z \phi_z\right) \mathrm{d}V. \end{aligned} \tag{S.6}$$

Based on the orthogonality between the normal modes, the last term on the right side of the equation (S.6) vanishes. By defining effective inertial masses for the normal modes

$$m_1 = \iiint\limits_V \rho \left(\psi_x^2 + \psi_y^2 + \psi_z^2\right) \mathrm{d}V, \qquad m_2 = \iiint\limits_V \rho \left(\phi_x^2 + \phi_y^2 + \phi_z^2\right) \mathrm{d}V, \tag{S.7}$$

one can simplify T_1 as

$$T_1 = \frac{1}{2} m_1 \dot{q}_1^2 + \frac{1}{2} m_2 \dot{q}_2^2. \tag{S.8}$$

Expanding T_2 we have

$$
\begin{aligned}
T_2 = &\frac{1}{2}\Omega^2 q_1^2 \rho \iiint\limits_V \left(\psi_x^2 + \psi_y^2\right) \mathrm{d}V + \frac{1}{2}\Omega^2 q_2^2 \rho \iiint\limits_V \left(\phi_x^2 + \phi_y^2\right) \mathrm{d}V \\
&+ \Omega^2 q_1 q_2 \rho \iiint\limits_V \left(\psi_x \phi_x + \psi_y \phi_y\right) \mathrm{d}V \\
&+ \Omega^2 q_1 \rho \iiint\limits_V \left(x\psi_x + y\psi_y\right) \mathrm{d}V + \Omega^2 q_2 \rho \iiint\limits_V \left(x\phi_x + y\phi_y\right) \mathrm{d}V.
\end{aligned} \tag{S.9}
$$

The T_2 is responsible for the centrifugal force, which only concerns the projection into the x-y plane of the resonator. Based on the orthogonality between normal modes, the third term on the right side of the equation (S.9) vanishes. The first and second terms are the effective centrifugal terms inside the normal modes. The centrifugal force produced is only proportional to the amplitudes q_n and has nothing to do with the resonator geometry.

The last two terms are more interesting, which are responsible for the resonator-geometry-induced centrifugal forces acting on the normal modes. We denote the sum of them by $T_{\mathrm{c,geom}}$. To further reveal the physical picture of those terms, we assume the resonator vibrates in the x-y plane, which is true for our disk resonator. The centrifugal forces acting on mode n ($n = 1, 2$) that are produced by $T_{\mathrm{c,geom}}$ can be derived as

$$
f_{\mathrm{c,geom}} = \frac{\partial T_{\mathrm{c,geom}}}{\partial q_n} = \Omega^2 \rho \int_{-H}^{0} \int_{0}^{2\pi} \int_{0}^{R} \mathbf{s} \cdot \boldsymbol{\psi} \mathrm{d}r \mathrm{d}\theta \mathrm{d}z. \tag{S.10}
$$

Here, the volume integration is described in a cylindrical-coordinate system r-θ-z; r is the radial coordinate, and θ is the azimuthal coordinate. R and H are the radius and height of the disk resonator, respectively; q_n and $\boldsymbol{\psi}$ are the amplitude and the shape function of the mode, respectively. Instead of decomposing $\boldsymbol{\psi}$ along the x and y directions, we decompose $\boldsymbol{\psi}$ into its tangential part $\boldsymbol{\psi}_{\mathrm{t}}$ and radial part $\boldsymbol{\psi}_{\mathrm{r}}$. Because $\mathbf{s} \cdot \boldsymbol{\psi}_{\mathrm{t}} = 0$, we have

$$
f_{\mathrm{c,geom}} = \Omega^2 \rho \int_{-H}^{0} \int_{0}^{2\pi} \int_{0}^{R} \mathbf{s} \cdot \boldsymbol{\psi}_{\mathrm{r}} \mathrm{d}r \mathrm{d}\theta \mathrm{d}z = \Omega^2 \rho \int_{-H}^{0} \int_{0}^{2\pi} \int_{0}^{R} |\mathbf{s}||\boldsymbol{\psi}_{\mathrm{r}}| \mathrm{d}r \mathrm{d}\theta \mathrm{d}z. \tag{S.11}
$$

This centrifugal force is produced by the rotating resonator body but acting on a dedicated mode; thus, the radius of each mass point is normalized by the radial component of the shape function of the mode. For the symmetric disk resonator, the overall effect of this centrifugal force is proportional to the integration of the mode shape function. For some kinds of modes, such as the breathing mode for disks or rings and the anti-phase mode for the tuning fork resonators, the effect of the geometry-induced centrifugal force acting on them cannot be neglected. However, for our four-node normal modes, there are nodes and antinodes. The integration of the mode shape function is zero. The efficiency of the geometry-induced centrifugal

force acting on the four-node mode is 0%. Thus the last two terms on the right side of the equation (S.9) vanish.

By defining inertial parameters

$$n_1 = \iiint_V \rho\left(\psi_x^2 + \psi_y^2\right) \mathrm{d}V, \qquad n_2 = \iiint_V \rho\left(\phi_x^2 + \phi_y^2\right) \mathrm{d}V, \tag{S.12}$$

one can simplify T_2 as

$$T_2 = \frac{1}{2}\Omega^2 n_1 q_1^2 + \frac{1}{2}\Omega^2 n_2 q_2^2. \tag{S.13}$$

Expanding T_3 and neglecting the position elements x and y because they will not produce inertial force, we have

$$T_3 = \Omega\left(q_1\dot{q}_2 - \dot{q}_1 q_2\right) \iiint_V \rho\left(\psi_x\phi_y - \psi_y\phi_x\right) \mathrm{d}V. \tag{S.14}$$

By defining an effective inertial parameter

$$m_\mathrm{c} = \iiint_V \rho\left(\psi_x\phi_y - \psi_y\phi_x\right) \mathrm{d}V. \tag{S.15}$$

one can simplify T_3 as

$$T_3 = \Omega m_\mathrm{c}\left(q_1\dot{q}_2 - \dot{q}_1 q_2\right). \tag{S.16}$$

By combining equations (S.8), (S.13), and (S.16), the kinetic energy of the rotating system (S.2) can be rewritten as

$$T = \frac{1}{2}m_1\dot{q}_1^2 + \frac{1}{2}m_2\dot{q}_2^2 + \Omega m_\mathrm{c}\left(q_1\dot{q}_2 - \dot{q}_1 q_2\right) + \frac{1}{2}\Omega^2 n_1 q_1^2 + \frac{1}{2}\Omega^2 n_2 q_2^2. \tag{S.17}$$

The potential energy $U(\mathbf{r})$ is mostly contributed by the mechanical springs, which can be expressed in the form

$$U(x, y) = \frac{1}{2}m_1\omega_1^2 q_1^2 + \frac{1}{2}m_2\omega_2^2 q_2^2, \tag{S.18}$$

where ω_1 and ω_2 are the effective angular frequencies of the two normal modes. The hybrid stiffness coupling between modes 1 and 2 is largely eliminated beforehand [7] and not considered here.

Based on equations (S.17) and (S.18), the Lagrangian of the vibrating resonator in the rotating frame is given by

$$\begin{aligned} L = T - U = {} & \frac{1}{2}m_1\dot{q}_1^2 + \frac{1}{2}m_2\dot{q}_2^2 + \Omega m_\mathrm{c}\left(q_1\dot{q}_2 - \dot{q}_1 q_2\right) + \frac{1}{2}\Omega^2\left(n_1 q_1^2 + n_2 q_2^2\right) \\ & - \frac{1}{2}m_1\omega_1^2 q_1^2 - \frac{1}{2}m_2\omega_2^2 q_2^2. \end{aligned} \tag{S.19}$$

The momenta conjugate to the displacements q_1 and q_2 are

$$p_1 = \frac{\partial L}{\partial \dot{q_1}} = m_1 \dot{q_1} - m_c \Omega q_2, \tag{S.20}$$

$$p_2 = \frac{\partial L}{\partial \dot{q_2}} = m_2 \dot{q_2} + m_c \Omega q_1, \tag{S.21}$$

respectively. The Hamiltonian of the rotating system can be derived from the Lagrangian (S.19) using the Legendre transformation,

$$\begin{aligned} H = &\frac{1}{2m_1}(p_1 + m_c \Omega q_2)^2 + \frac{1}{2m_2}(p_2 - m_c \Omega q_1)^2 - \frac{1}{2}\Omega^2 \left(n_1 q_1^2 + n_2 q_2^2\right) \\ &+ \frac{1}{2} m_1 \omega_1^2 q_1^2 + \frac{1}{2} m_2 \omega_2^2 q_2^2. \end{aligned} \tag{S.22}$$

The additional $m_c \Omega q_{1,2}$ terms are the effective magnetic fields.

Then, we define the bosonic variables b_1 and b_2,

$$b_{1,2} = \frac{1}{\sqrt{2\hbar}} \left(\sqrt{m_{1,2}\omega_{1,2}} q_{1,2} + \frac{\mathrm{i} p_{1,2}}{\sqrt{m_{1,2}\omega_{1,2}}} \right), \tag{S.23}$$

where $\hbar$ is a constant number, which we can consider it as $\hbar = 1$. We can rewrite the Hamiltonian in quadratic form,

$$\begin{aligned} H = &\hbar\omega_1 b_1^* b_1 + \hbar\omega_2 b_2^* b_2 + \frac{\hbar}{2}\left(\mathrm{i}\eta'\kappa\Omega b_1^* b_2^* + \text{c.c.}\right) + \frac{\hbar}{2}\left(\mathrm{i}\eta\kappa\Omega b_1^* b_2 + \text{c.c.}\right) \\ &+ \frac{\hbar\Omega^2}{4\omega_1}\left(\kappa^2 - \kappa_1'\right)\left(b_1 + b_1^*\right)^2 + \frac{\hbar\Omega^2}{4\omega_2}\left(\kappa^2 - \kappa_2'\right)\left(b_2 + b_2^*\right)^2. \end{aligned} \tag{S.24}$$

Here, c.c. denotes the complex conjugate of the preceding term,

$$\kappa_j = m_c / m_j, \tag{S.25}$$

for $j = 1, 2$ are defined as the Coriolis coupling coefficients; $\kappa = \sqrt{\kappa_1 \kappa_2}$ is the mean coefficient of the Coriolis coupling, which is the measure of the efficiency of the vibrational energy transfer induced by the Coriolis force and is determined by the vibration mode-shapes. In this study, the effective masses of the degenerate normal modes are identical, which means $m = m_1 = m_2$ and $\kappa = \kappa_1 = \kappa_2$; $\kappa_j' = n_j / m_j$ are defined as the centrifugal coefficients; the parameters η and η' are given by $\eta = \sqrt{\omega_1/\omega_2} + \sqrt{\omega_2/\omega_1}$ and $\eta' = \sqrt{\omega_1/\omega_2} - \sqrt{\omega_2/\omega_1}$, respectively.

The state b_j oscillates at the frequency $-\omega_j$ and b_j^* oscillates at the frequency $+\omega_j$. By applying the rotating wave approximation, the fast oscillating terms $b_1^* b_2^*$ and $b_1 b_2$ in the Hamiltonian (S.24) are neglected. For the in-plane vibrating orthogonal modes of this study, the term $(\kappa^2 - \kappa_j')$ is zero, which can

be verified by checking $m_c = m_2 n_1 = m_1 n_2$. To sum up, the Hamiltonian of the rotating resonator can be further simplified as

$$H = \hbar\omega_1 b_1^* b_1 + \hbar\omega_2 b_2^* b_2 + \frac{\mathrm{i}}{2}\hbar\eta\kappa\Omega\left(b_1^* b_2 - b_2^* b_1\right). \tag{S.26}$$

The eigenfrequencies of the Coriolis coupled modes are further obtained by calculating the eigenvalues of the matrix

$$\begin{bmatrix} \omega_1 & \frac{1}{2}\mathrm{i}\eta\kappa\Omega \\ -\frac{1}{2}\mathrm{i}\eta\kappa\Omega & \omega_2 \end{bmatrix},$$

which are given by

$$\omega_\pm = \frac{\omega_1 + \omega_2}{2} \pm \frac{1}{2}\sqrt{\Delta\omega^2 + \eta^2\kappa^2\Omega^2}. \tag{S.27}$$

where $\Delta\omega = \omega_2 - \omega_1$ is the frequency difference. By calculating the degenerate frequency split, we obtain the coupling strength $g = 2\kappa\Omega$.

SUPPLEMENTARY NOTE 3. PHASE-TRACKED CLOSED-LOOP OSCILLATION

A. Equations of motion and open-loop responses

In this study, the steady state under continuous driving is considered. More specifically, we are interested in those steady states whose phases are tracked to the drive reference in quadrature, or the so-called phase-tracked closed-loop oscillation. In this section, we derive the theoretical model for this closed-loop oscillation. We start by considering a drive on mode 1, and the Hamiltonian is further given by

$$H_\mathrm{d} = \hbar\omega_1 b_1^* b_1 + \hbar\omega_2 b_2^* b_2 + \frac{\mathrm{i}}{2}\hbar\eta\kappa\Omega\left(b_1^* b_2 - b_2^* b_1\right) - \hbar f_0 (b_1 \mathrm{e}^{\mathrm{i}\omega_\mathrm{d} t} + b_1^* \mathrm{e}^{-\mathrm{i}\omega_\mathrm{d} t}). \tag{S.28}$$

where f_0 is the normalized force defined by $\frac{1}{2}F_0(2\hbar m\omega_1)^{-1/2}$.

We use the Langevin equations of motion for the bosonic variables b_j^* ($j = 1, 2$) to describe this system,

$$\frac{\mathrm{d}}{\mathrm{d}t} b_j^* = \frac{\mathrm{i}}{\hbar}\{H_\mathrm{d}, b_j^\dagger\} - \frac{\gamma}{2} b_j^* + \xi_j^*. \tag{S.29}$$

where γ is the dissipation rate of the degenerate modes and ξ_j^* indicates the thermal Langevin force acting on the mode b_j^*. Here, we give the reason why we do not use the Langevin equations about the variables b_j as usual. In the complex plane, b_j (b_j^*) rotates in the clockwise (counter-clockwise) direction with a phase factor $\mathrm{e}^{-\mathrm{i}\omega_j t}$ ($\mathrm{e}^{\mathrm{i}\omega_j t}$). In this work, the counter-clockwise direction is defined as the positive rotation direction. The equations of motion about the variables b_j would result in a counter-intuitive scenario

that a positive rotation applied upon a counter-clockwise circular polarized travelling-wave state produces frequency reduction, which is the case if the polarization directions of the two stable bifurcation branches in Fig. 2g and Fig. 3b of the main text are exchanged. Equations about the variables b_j^* will perfectly mend this error.

In this study, we are only interested in the coherent responses about the external drive, and the fluctuation terms are neglected, the equations of motion can be obtained,

$$\begin{bmatrix} \dot{b}_1^* \\ \dot{b}_2^* \end{bmatrix} = \begin{bmatrix} \mathrm{i}\omega_1 - \frac{1}{2}\gamma & \frac{1}{2}\eta\kappa\Omega \\ -\frac{1}{2}\eta\kappa\Omega & \mathrm{i}\omega_2 - \frac{1}{2}\gamma \end{bmatrix} \begin{bmatrix} b_1^* \\ b_2^* \end{bmatrix} - \begin{bmatrix} \mathrm{i}f_0 \\ 0 \end{bmatrix} \mathrm{e}^{\mathrm{i}\omega_\mathrm{d}t}. \tag{S.30}$$

To obtain the steady-state responses, we transform the system into a frame rotating at the driving frequency ω_d by writing $b_{1,2}^* = \alpha_{1,2}\mathrm{e}^{\mathrm{i}\omega_\mathrm{d}t}$. The equations of motion about the complex amplitudes $\alpha_{1,2}$ in the rotating frame are given by

$$\begin{bmatrix} \dot{\alpha}_1 \\ \dot{\alpha}_2 \end{bmatrix} = \begin{bmatrix} -\mathrm{i}\Delta_1 - \frac{1}{2}\gamma & \frac{1}{2}\eta\kappa\Omega \\ -\frac{1}{2}\eta\kappa\Omega & -\mathrm{i}\Delta_2 - \frac{1}{2}\gamma \end{bmatrix} \begin{bmatrix} \alpha_1 \\ \alpha_2 \end{bmatrix} - \begin{bmatrix} \mathrm{i}f_0 \\ 0 \end{bmatrix}, \tag{S.31}$$

where $\Delta_{1,2} = \omega_\mathrm{d} - \omega_{1,2}$ denote the driving detunings. Here, we consider the steady states that can be obtained by applying $\dot{\alpha}_{1,2} = 0$, which gives $\alpha_{1,2} = f_0\chi_{1,2}(\omega_\mathrm{d})$. The mechanical susceptibilities $\chi_{1,2}$ are given by

$$\begin{aligned} \chi_1(\omega_\mathrm{d}) &= \frac{\omega_\mathrm{d} - \omega_2 - \mathrm{i}\gamma/2}{[\mathrm{i}(\omega_\mathrm{d} - \omega_1) + \gamma/2]\,[\mathrm{i}(\omega_\mathrm{d} - \omega_2) + \gamma/2] + \eta^2\kappa^2\Omega^2/4} \\ &= \frac{\omega_2 - \omega_\mathrm{d} + \mathrm{i}\gamma/2}{(\omega_\mathrm{d} - \omega_+ - \mathrm{i}\gamma/2)(\omega_\mathrm{d} - \omega_- - \mathrm{i}\gamma/2)}, \end{aligned} \tag{S.32}$$

$$\begin{aligned} \chi_2(\omega_\mathrm{d}) &= \frac{\mathrm{i}\eta\kappa\Omega/2}{[\mathrm{i}(\omega_\mathrm{d} - \omega_1) + \gamma/2]\,[\mathrm{i}(\omega_\mathrm{d} - \omega_2) + \gamma/2] + \eta^2\kappa^2\Omega^2/4} \\ &= \frac{-\mathrm{i}\eta\kappa\Omega/2}{(\omega_\mathrm{d} - \omega_+ - \mathrm{i}\gamma/2)(\omega_\mathrm{d} - \omega_- - \mathrm{i}\gamma/2)}, \end{aligned} \tag{S.33}$$

where $\omega_\pm$ are the eigenfrequencies given by (S.27).

Based on the relations $q_{1,2} = \left(b_{1,2} + b_{1,2}^*\right)/\sqrt{2m\omega_{1,2}}$, the steady-state mechanical displacements that can be experimentally measured are given by

$$\begin{aligned} q_1(\omega_\mathrm{d}, t) &= [\chi_1(\omega_\mathrm{d})\mathrm{e}^{\mathrm{i}\omega_\mathrm{d}t} + \mathrm{c.c.}]F_0/(4m\omega_1), \\ q_2(\omega_\mathrm{d}, t) &= [\chi_2(\omega_\mathrm{d})\mathrm{e}^{\mathrm{i}\omega_\mathrm{d}t} + \mathrm{c.c.}]F_0/(4m\sqrt{\omega_1\omega_2}). \end{aligned}$$

By writing $q_{1,2} = |q_{1,2}|\cos(\omega_\mathrm{d}t + \theta_{1,2})$, we obtain the frequency responses. The theoretical amplitude-

frequency responses are given by

$$|q_1| = \frac{F_0}{2m\omega_1}|\chi_1|, \tag{S.34}$$

$$|q_2| = \frac{F_0}{2m\sqrt{\omega_1\omega_2}}|\chi_2|. \tag{S.35}$$

The theoretical phase-frequency responses $\theta_{1,2}(\omega_\mathrm{d})$ are given by the phase angles $\angle\chi_{1,2}$ of the susceptibilities in the complex plane,

$$\theta_1 = \angle\chi_1, \tag{S.36}$$

$$\theta_2 = \angle\chi_2. \tag{S.37}$$

The experimentally measured frequency responses in Supplementary Fig. **4** are simulated based on (S.34)-(S.37), as shown in Supplementary Fig. **5**. The amplitude-frequency responses $|q_{1,2}|(\omega_\mathrm{d})$ are simulated by calculating the moduli of the susceptibilities multiplied by a fitting factor, $c|\chi_{1,2}|$. The factor $c = 8.6\times10^{-3}$ is related to the drive and transduction efficiencies.

B. Phase-tracked oscillation

To better reveal the phase-tracked closed-loop oscillation, which is defined as the steady state whose driven-mode phase is kept in quadrature to the drive reference ($\theta_1 = -\pi/2$), we draw the polar plots of the two quadrature components of the driven mode 1 in the complex plane, as shown in Supplementary Fig. **6**. The in-phase (quadrature) component $|q_1|\cos\theta_1$ ($|q_1|\sin\theta_1$) is fitted by the real (imaginary) part of the susceptibility $c\Re(\chi_1)$ ($c\Im(\chi_1)$), with the fitting parameter $c = 8.6\times10^{-3}$ given previously. The amplitude $|q_1|$ is represented by the distance between the point on the polar plot to the origin, and the phase θ_1 is represented by the phase angle $\angle\chi_1$ in the complex plane polar plot. The $\theta_1 = -\pi/2$ phase-tracked steady states are shown by the intersection points of the polar plots and the negative half imaginary axis in Supplementary Fig. **6**. The corresponding driving frequency satisfying the $\theta_1 = -\pi/2$ phase-tracked condition ω_d^* can be calculated by eliminating the real part of the mode-1 susceptibility, $\Re[\chi_1(\omega_\mathrm{d}^*)] = 0$, which is given by a cubic equation about ω_d^*:

$$(\omega_\mathrm{d}^* - \omega_1)(\omega_\mathrm{d}^* - \omega_2 + \frac{\mathrm{i}}{2}\gamma)(\omega_\mathrm{d}^* - \omega_2 - \frac{\mathrm{i}}{2}\gamma) - \frac{1}{4}\eta^2\kappa^2\Omega^2(\omega_\mathrm{d}^* - \omega_2) = 0. \tag{S.38}$$

The above equation can fully simulate the folded surface in Fig. 1d of the main text. It can also be rewritten in another form,

$$(\omega_\mathrm{d}^* - \omega_2)(\omega_\mathrm{d}^* - \omega_+)(\omega_\mathrm{d}^* - \omega_-) + \frac{\gamma^2}{4}(\omega_\mathrm{d}^* - \omega_1) = 0, \tag{S.39}$$

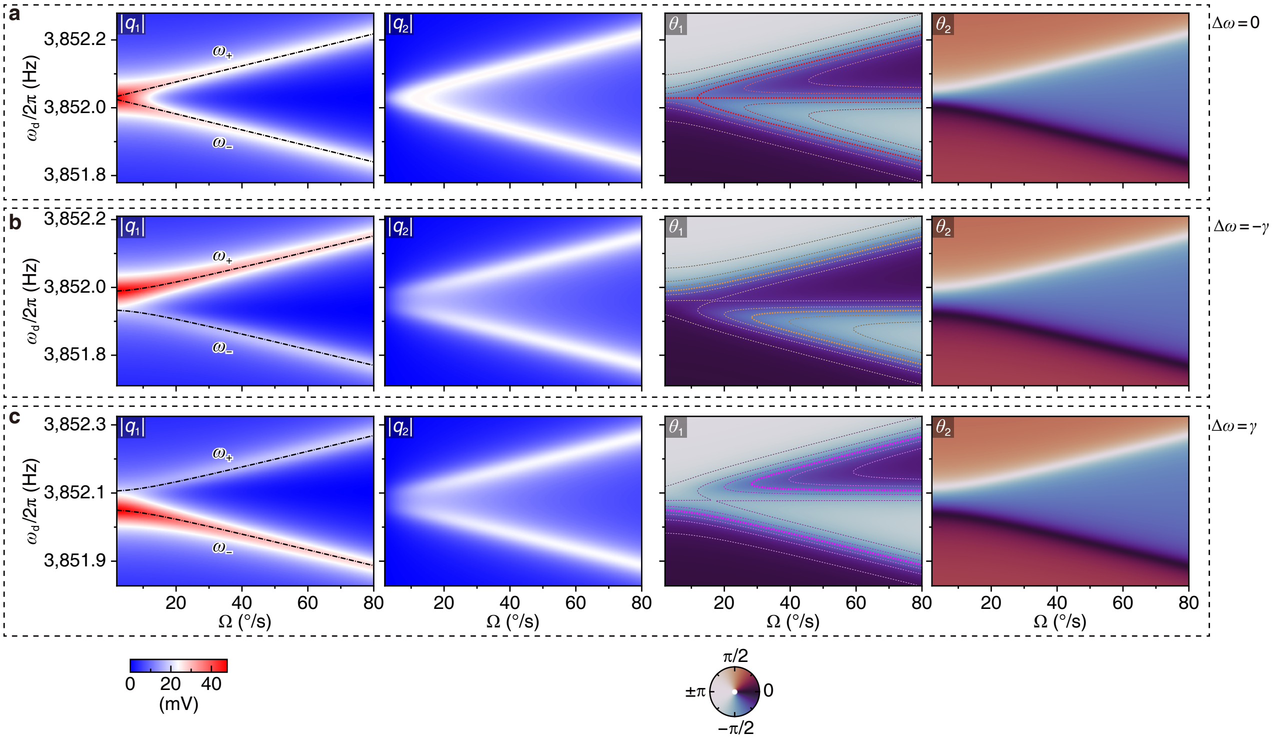

Supplementary Figure 5. **Simulated frequency responses corresponding to the experimental data in Supplementary Fig. 4.** (**a**), In the mode-matched case, $\Delta\omega = 0$, simulated frequency responses of the amplitudes $|q_{1,2}|(\omega_\mathrm{d})$ and phases $\theta_{1,2}(\omega_\mathrm{d})$ as functions of the angular velocity Ω. (**b**) Same as (a) for the case of $\Delta\omega = -\gamma$. (**c**) Same as (a) for the case of $\Delta\omega = \gamma$. In the $|q_1|(\omega_\mathrm{d})$ responses, the eigenfrequencies $\omega_\pm$ as functions of Ω are marked by the black dot-dashed lines. In the $\theta_1(\omega_\mathrm{d})$ responses, the phase-tracked equiphase contours of $\theta_1 = \{-\pi/8, -\pi/4, -3\pi/8, -\pi/2, -5\pi/8, -3\pi/4, -7\pi/8\}$ are drawn by dotted curves.

where $\omega_\pm$ are the eigenfrequencies of the Coriolis coupled modes given by (S.27). It is the dissipation term, the second component on the left side of equation (S.39), that causes the singularity. In the absence of dissipation, the phase-tracked frequency in the parameter space (grey surfaces in Supplementary Fig. **7**a) takes the form of two diabolical cones ($\omega_\pm$) connected by a tangential plane (ω_2), and the folding vanishes into a red intersecting line. As dissipation arises, the ω_d^* surface splits from the intersection line, leading to the orange, partially folded geometry in Supplementary Fig. **7**b.

C. "Pitchfork" bifurcation and singularity nexus

The phase-tracked frequencies ω_d^* as functions of the angular velocity Ω at different degeneracy conditions are calculated based on (S.39) and are shown by the red contours in the θ_1 responses of Supplementary Fig. **4**, respectively, which depicts bifurcation patterns. Here, we consider the "pitchfork" bifurcation, which

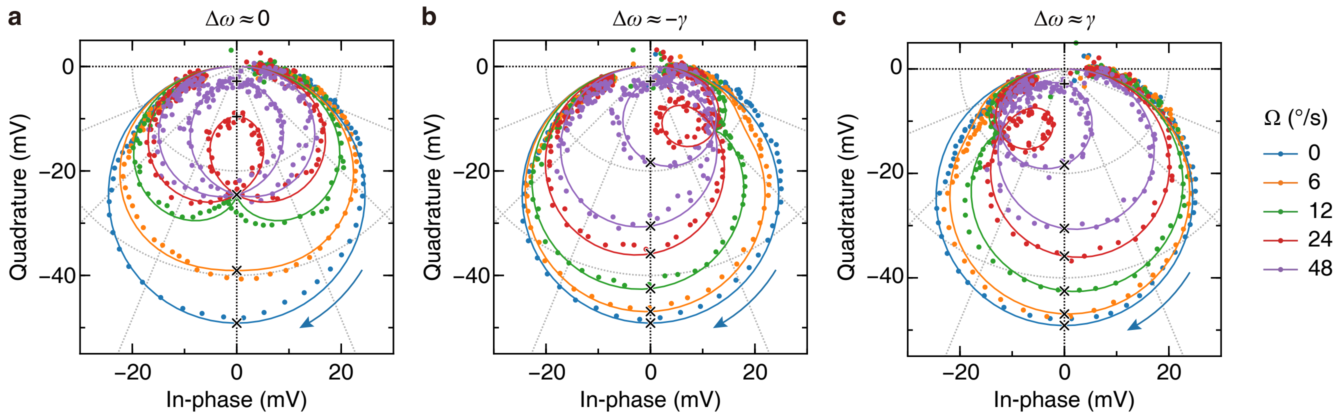

Supplementary Figure 6. **Polar plots of the driven-mode responses.** Polar plots of q_1 as functions of Ω for degeneracy conditions of $\Delta\omega = 0$ (**a**), $\Delta\omega = -\gamma$ (**b**), and $\Delta\omega = \gamma$ (**c**). The in-phase (quadrature) part of the experimental data (points) indicates $|q_1|\cos\theta_1$ ($|q_1|\sin\theta_1$), which is fitted by the real (imaginary) part of the driven mode susceptibility $c\Re[\chi_1]$ ($-c\Im[\chi_1]$) with parameter $c = 8.6 \times 10^{-3}$. The $\theta_1 = -\pi/2$ phase-tracked closed-loop states are represented by the intersections between the polar curves and the negative-imaginary axis. The intersection points marked by "×" ("+") are stable (unstable).

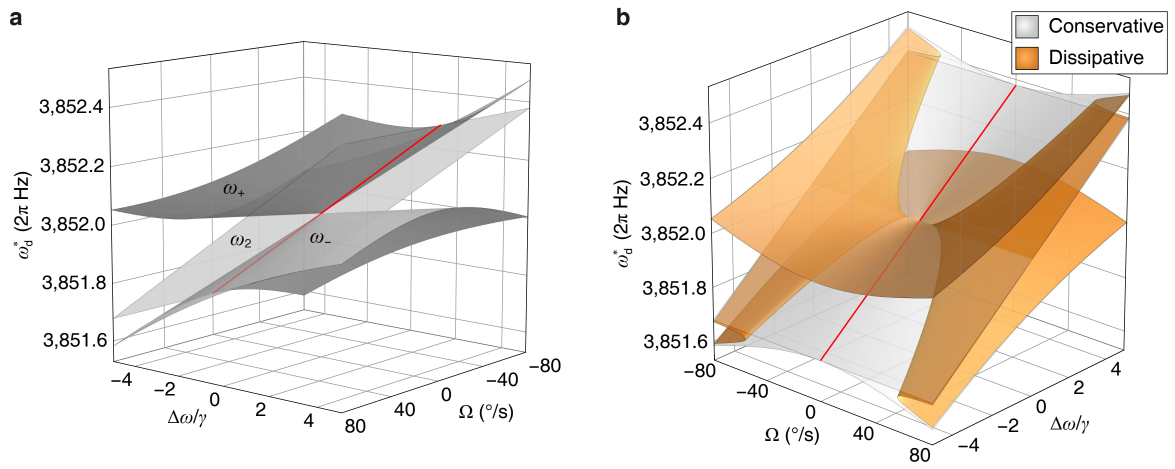

Supplementary Figure 7. **Dissipation dependency of the phase-tracked singularity.** (**a**) If $\gamma \to 0$, the ω_d^* surface becomes two diabolical cones ($\omega_\pm$) connected by a tangential plane (ω_2). (**b**) If dissipation arises, the surface splits from the intersection (red) line and singularity emerges.

is the case when perfect degeneracy is fulfilled. By applying the degeneracy condition, $\omega_1 = \omega_2 = \omega_0$, the cubic equation (S.39) about ω_d^* reduces to a factorized form

$$(\omega_\mathrm{d}^* - \omega_0)\left[(\omega_\mathrm{d}^* - \omega_+)(\omega_\mathrm{d}^* - \omega_-) + \frac{\gamma^2}{4}\right] = 0.$$

The discriminant of the quadratic equation $(\omega_\mathrm{d}^* - \omega_+)(\omega_\mathrm{d}^* - \omega_-) + \gamma^2/4 = 0$ determines how many real solutions exist. If the quadratic discriminant is negative, $|\Omega| < \gamma/(2\kappa)$, the only solution is $\omega_\mathrm{d}^* = \omega_0$. If the quadratic discriminant is positive, $|\Omega| > \gamma/(2\kappa)$, there are three solutions. Later, we will show that the middle one $\omega_\mathrm{d}^* = \omega_0$ is unstable and the other two solutions are stable. Therefore, by equating the discriminant of the quadratic equation to zero, we obtain the "pitchfork" bifurcation point latter referred to as singularity nexus, which is calculated to be

$$|\Omega_0| = \frac{\gamma}{2\kappa}. \tag{S.40}$$

SUPPLEMENTARY NOTE 4. STABILITY ANALYSIS

Each point on the partially folded 3D surface in Fig. 1d of the main text represents a PhT steady state. Here, we analyse the stabilities of the PhT steady states and reveal that the inflectional part of the folded surface is unstable. We start with the equations of motion (S.31) and rewrite the complex amplitudes in the form of $\alpha_{1,2} = f_0|\chi_{1,2}|\mathrm{e}^{\theta_{1,2}}$, where $\chi_{1,2}, \theta_{1,2} \in \mathbb{R}$ are the normalized amplitudes and phases. By applying the phase-tracking condition $\theta_1 = -\pi/2$, the steady dynamics of the PhT states are given by $\mathbf{F}(|\chi_1|, |\chi_2|, \theta_2, \omega_\mathrm{d}^*) = \mathbf{0}$,

$$1 - \frac{\gamma}{2}|\chi_1| - \frac{1}{2}\eta\kappa\Omega|\chi_2|\sin\theta_2 = 0, \tag{S.41}$$

$$\frac{1}{2}\eta\kappa\Omega|\chi_1|\sin\theta_2 - \frac{\gamma}{2}|\chi_2| = 0, \tag{S.42}$$

$$\frac{1}{2}\eta\kappa\Omega|\chi_1|\cos\theta_2 - (\omega_\mathrm{d}^* - \omega_2)|\chi_2| = 0, \tag{S.43}$$

$$\frac{1}{4}\eta^2\kappa^2\Omega^2(\omega_\mathrm{d}^* - \omega_2) - (\omega_\mathrm{d}^* - \omega_1)\big[(\omega_\mathrm{d}^* - \omega_2)^2 + \frac{\gamma^2}{4}\big] = 0, \tag{S.44}$$

Based on the Hartman–Grobman theorem [11], the stabilities of the PhT steady states are determined by the real eigenvalues of the Jacobian matrix of $\mathbf{F}(|\chi_1|, |\chi_2|, \theta_2, \omega_\mathrm{d}^*)$,

$$\mathbf{J} = \begin{bmatrix} -\frac{\gamma}{2} & -\frac{1}{2}\eta\kappa\Omega\sin\theta_2 & -\frac{1}{2}\eta\kappa\Omega|\chi_2|\cos\theta_2 & 0 \\ \frac{1}{2}\eta\kappa\Omega\sin\theta_2 & -\frac{\gamma}{2} & \frac{1}{2}\eta\kappa\Omega|\chi_1|\cos\theta_2 & 0 \\ \frac{1}{2}\eta\kappa\Omega\cos\theta_2 & \omega_2 - \omega_\mathrm{d}^* & -\frac{1}{2}\eta\kappa\Omega|\chi_1|\sin\theta_2 & -|\chi_2| \\ 0 & 0 & 0 & \frac{1}{4}\eta^2\kappa^2\Omega^2 - \frac{\gamma^2}{4} - (\omega_\mathrm{d}^* - \omega_2)(3\omega_\mathrm{d}^* - 2\omega_1 - \omega_2) \end{bmatrix}, \tag{S.45}$$

which describes the divergence or convergence of the system near the fixed points (PhT steady states) with small perturbations. If the real parts of all the Jacobian eigenvalues are negative, the perturbed system near the fixed point is convergent. In this case, the phase-tracked state is stable. Otherwise, if any of the

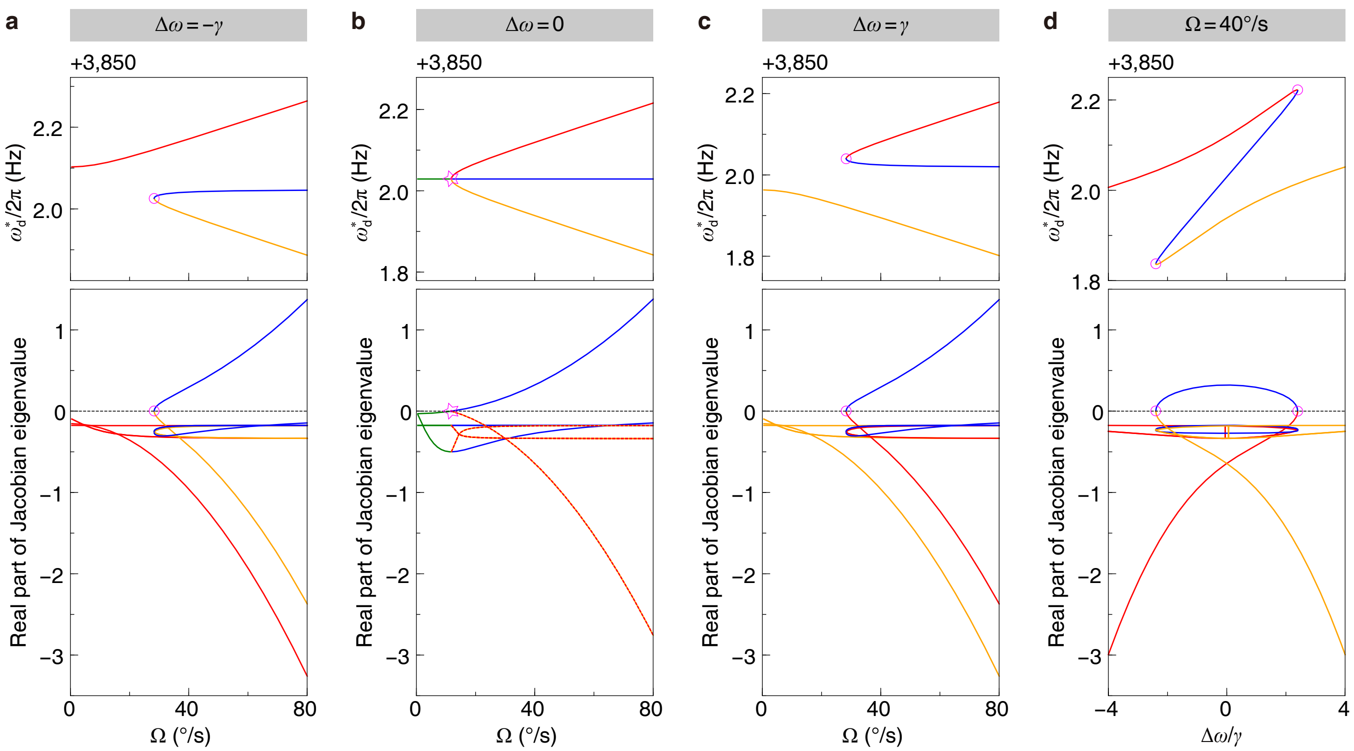

Supplementary Figure 8. Calculated real Jacobian eigenvalues of different PhT states for (**a**) $\Delta\omega = -\gamma$, (**b**) $\Delta\omega = 0$, (**c**) $\Delta\omega = \gamma$, and (**d**) $\Omega = 40°/\mathrm{s}$. The upper panels are the PhT frequencies. The corresponding real parts of the Jacobian eigenvalues are shown in the lower panels. The real parts of Jacobian eigenvalues of the middle branch (inflectional part) marked by blue colour have positive values, which indicate unstable vibrations.

Jacobian eigenvalues have positive or zero real parts, the phase-tracked state will be unstable or critically stable, respectively. The critically stable phase-tracked states are referred to as the singularities.

The calculated Jacobian eigenvalue real parts of some typical contours of the partially folded surface of ω_d^* are shown in Supplementary Fig. **8**. The upper panels are the PhT frequencies and the lower panels are the calculated real parts of the Jacobian eigenvalues of the corresponding PhT frequencies (states) marked by identical colours. As shown in Supplementary Fig. **8**a-c, the middle branch of each bifurcation (blue part) is unstable, while the other (red orange, green) parts are stable. The bifurcation points act as transition points between stable and unstable states, which turn out to be the singularities. As shown in Supplementary Fig. **8**d, the inflectional (blue) part (which is made up of the middle bifurcation branch) is unstable. By analyzing the stabilities of the PhT states on the ω_d^* surface in Fig. 1d of the main text, the stable and unstable parts are marked by light green and red, respectively.

SUPPLEMENTARY NOTE 5. CUBIC SINGULARITY

The cubic equation of the phase-tracked frequency (S.39) gives a partially folded geometry. The singularity is produced by the projection of the folding surface to the parameter plane, which is given by the stability boundaries. The discriminant of (S.39) determines how many real solutions of the phase-tracked frequencies exist, thus the singularities. We expand the cubic equation (S.39) in the following form

$$\omega_{\mathrm{d}}^{*3} + A_2\omega_{\mathrm{d}}^{*2} + A_1\omega_{\mathrm{d}}^{*} + A_0 = 0,$$

where

$$\begin{aligned} A_2 &= -\omega_1 - 2\omega_2, \\ A_1 &= 2\omega_1\omega_2 + \omega_2^2 - \eta^2\kappa^2\Omega^2/4 + \gamma^2/4, \\ A_0 &= -\omega_1\omega_2^2 + \omega_2\eta^2\kappa^2\Omega^2/4 - \omega_1\gamma^2/4. \end{aligned}$$

By equating the discriminant of the cubic equation (S.39) to zero, $4(3A_1-A_2^2)^3+(2A_2^3-9A_2A_1+27A_0)^2 = 0$, we obtain the singularities at which discontinuous jumps might occur. The discriminant equation can be further simplified as

$$16\gamma^2\Delta\omega^4 + (8\gamma^4 + 20\eta^2\kappa^2\Omega^2\gamma^2 - \eta^4\kappa^4\Omega^4)\Delta\omega^2 - (\eta^2\kappa^2\Omega^2 - \gamma^2)^3 = 0, \tag{S.46}$$

which is a quadratic equation about $\Delta\omega^2$. By solving this equation, we obtain the singularities explicitly in the Ω-$\Delta\omega$ plane (Cyan curves in Fig. 1d of the main text):

$$\Delta\omega^2 = \frac{\eta^4\kappa^4\Omega^4}{32\gamma^2} - \frac{5\eta^2\kappa^2\Omega^2}{8} - \frac{\gamma^2}{4} \pm \sqrt{\frac{\eta^8\kappa^8\Omega^8}{32^2\gamma^4} + \frac{3\eta^6\kappa^6\Omega^6}{128\gamma^2} + \frac{3\eta^4\kappa^4\Omega^4}{16} + \frac{\eta^2\kappa^2\Omega^2\gamma^2}{2}}. \tag{S.47}$$

By substituting the relationship between V_{t} and $\Delta\omega$, equation (3) of the Methods, into (S.47), we can calculate the singularities explicitly in the Ω-V_{t} plane (Fig. 2i of the main text). The cusp point (singularity nexus) $|\Omega_0| = \gamma/(2\kappa)$ can also be obtained by directly applying the degeneracy condition $\Delta\omega = 0$ to the equation (S.47).

The singularities (stability boundaries) projected to the Bloch sphere are shown in Fig. 3a of the main text, which are exactly the combinations of the bifurcation points in Supplementary Fig. **9**c. If the singularity curves are crossed, the catastrophe effect occurs, and the closed-loop oscillation state will discontinuously jump from the magenta unstable boundary to the green bistable boundary.

SUPPLEMENTARY NOTE 6. CLASSICAL BLOCH SPHERE

The coupled dynamics of the phase-tracked oscillation can be fully characterized by a state vector defined as $|\psi\rangle = (\alpha_1, \alpha_2)^{\mathrm{T}} = \alpha_1|1\rangle + \alpha_2|2\rangle$, where $\alpha_{1,2} = f_0\chi_{1,2}(\omega_{\mathrm{d}})$ are the steady-state complex amplitudes in the rotating frame. Here, $|1\rangle = (1,0)^{\mathrm{T}}$ and $|2\rangle = (0,1)^{\mathrm{T}}$ represent the state vectors of the eigenstates 1 and 2, respectively, which form an orthonormal basis $\{|1\rangle, |2\rangle\}$. By substituting the expressions of the mechanical susceptibilities $\chi_{1,2}$ (S.32) and (S.33) into $|\psi\rangle$, we can further expand the state vector

$$|\psi\rangle = f_0\sqrt{|\chi_1|^2 + |\chi_2|^2}\mathrm{e}^{\mathrm{i}\theta_1}\left(\cos\frac{\phi}{2}|1\rangle + \mathrm{e}^{\mathrm{i}\vartheta}\sin\frac{\phi}{2}|2\rangle\right),$$

where the polar angle is defined by

$$\phi = 2\arctan\frac{|\chi_2|}{|\chi_1|} = 2\arctan\frac{\eta k|\Omega|/2}{\sqrt{(\omega_{\mathrm{d}}^* - \omega_2)^2 + \gamma^2/4}}, \tag{S.48}$$

with $\phi \in [0, \pi]$. The relative phase is defined by

$$\vartheta = \theta_2 - \theta_1 = \arctan\frac{\omega_2 - \omega_{\mathrm{d}}^*}{\gamma/2}, \tag{S.49}$$

with $\vartheta \in [-\pi/2, 3\pi/2)$. By substituting the definition (S.49) in the phase-tracked frequency equation (S.39), one can obtain the direct expression of the relative phase of the $\theta_1 = -\pi/2$ phase-tracked oscillation, which is given by

$$\gamma^2\tan\vartheta - 2\Delta\omega\gamma - \eta^2\kappa^2\Omega^2\sin\vartheta\cos\vartheta = 0. \tag{S.50}$$

The coupled dynamics of the phase-tracked oscillation have gauge invariance, and in the following analyses, we can omit the global phase and normalize the state vector by writing

$$|\psi\rangle = \cos\frac{\phi}{2}|1\rangle + \mathrm{e}^{\mathrm{i}\vartheta}\sin\frac{\phi}{2}|2\rangle. \tag{S.51}$$

The state vectors described by (S.51) can be projected to a classic Bloch sphere shown in Supplementary Fig. **9**a. The state vectors in the sphere coordinates $\mathbf{S} = (\mathrm{S}_1, \mathrm{S}_2, \mathrm{S}_3)^{\mathrm{T}}$ are given by

$$\begin{aligned}
\mathrm{S}_1 &= \sin\phi\cos\vartheta = \frac{2|\chi_1||\chi_2|}{|\chi_1|^2 + |\chi_2|^2}\cos\vartheta = \frac{\eta k\Omega\gamma/2}{(\omega_{\mathrm{d}}^* - \omega_2)^2 + \gamma^2/4 + \eta^2k^2\Omega^2/4},\\
\mathrm{S}_2 &= \sin\phi\sin\vartheta = \frac{2|\chi_1||\chi_2|}{|\chi_1|^2 + |\chi_2|^2}\sin\vartheta = \frac{\eta k\Omega(\omega_2 - \omega_{\mathrm{d}}^*)}{(\omega_{\mathrm{d}}^* - \omega_2)^2 + \gamma^2/4 + \eta^2k^2\Omega^2/4},\\
\mathrm{S}_3 &= \cos\phi = \frac{|\chi_1|^2 - |\chi_2|^2}{|\chi_1|^2 + |\chi_2|^2} = \frac{(\omega_{\mathrm{d}}^* - \omega_2)^2 + \gamma^2/4 - \eta^2k^2\Omega^2/4}{(\omega_{\mathrm{d}}^* - \omega_2)^2 + \gamma^2/4 + \eta^2k^2\Omega^2/4}.
\end{aligned}$$

The S_1 parameter stands for the ellipticity of the polarization, which represents a circular (linear) orbit if $|\mathrm{S}_1| = 0$ ($|\mathrm{S}_1| = 1$). The S_2 parameter stands for the chirality of the polarization, which represents a

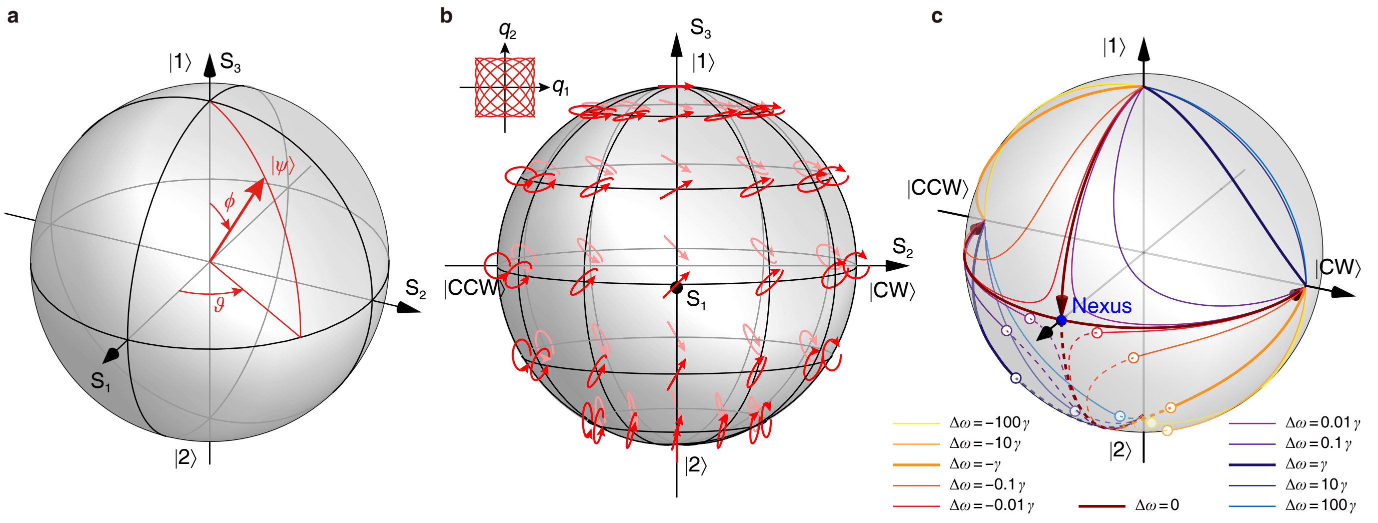

Supplementary Figure 9. Classical Bloch sphere describing the coupled dynamics of the phase-tracked oscillation. (**a**) The state vector $|\psi\rangle$ is projected to the Bloch sphere with polar angle ϕ and azimuthal angle ϑ. (**b**) Each point on the Bloch sphere corresponds to a polarization, which is revealed by the Lissajous pattern on the q_1-q_2 plane (Inset). (**c**) Phase-tracked state evolutions on the Bloch sphere with continuously changing Ω at constant values of $\Delta\omega$. Solid (dashed) curves indicate stable (unstable) states.

clockwise (counter-clockwise) orbit for a positive (negative) value of S_2. The S_3 parameter stands for the orientation of the polarization, or the relative population of the $|1\rangle$ and $|2\rangle$ states.

Each state vector corresponds to a Lissajous (polarization) pattern in the q_1-q_2 plane, indicated by the upper-left inset of Supplementary Fig. **9**b. The polarizations on the Bloch sphere are shown in Supplementary Fig. **9**b. The eigenstates $|1\rangle$ and $|2\rangle$ are located at the north pole $(0, 0, 1)^{\mathrm{T}}$ and south pole $(0, 0, -1)^{\mathrm{T}}$, respectively. The states located at $(0, 1, 0)^{\mathrm{T}}$ and $(0, -1, 0)^{\mathrm{T}}$, with clockwise and counter-clockwise circular polarizations, are defined as

$$|\mathrm{CW}\rangle = (|1\rangle + i\,|2\rangle)/\sqrt{2},$$
$$|\mathrm{CCW}\rangle = (|1\rangle - i\,|2\rangle)/\sqrt{2}, \tag{S.52}$$

respectively. The front-side (back-side) hemisphere corresponds to the states with positive (negative) values of Ω. The phase-tracked state evolutions on the front side of the Bloch sphere with continuously changing angular velocity with $\Omega \geq 0$ at constant values of $\Delta\omega$ are shown in Supplementary Fig. **9**c. The arrows indicate the Ω-increasing directions. For the perfect degeneracy condition, $\Delta\omega = 0$, the phase-tracked oscillation bifurcates in a "pitchfork" form at the singularity nexus $\Omega_0 = \gamma/(2\kappa)$ marked by a blue point located at $(1, 0, 0)^{\mathrm{T}}$ in Supplementary Fig. **9**c. The two stable bifurcation branches (solid curves) approaches circular-polarization states located at $(0, \pm 1, 0)^{\mathrm{T}}$ if $\Omega \to \infty$. The remaining unstable branch (dashed curve)

approximate to $(0,0,0)^{\mathrm{T}}$. If a mismatch between the natural frequencies of the two modes is introduced, the symmetry of the bifurcation breaks. In this case, one stable branch on the upper hemisphere directly goes from $|1\rangle$ to one of the two circular-polarization states, while the other two branches on the lower hemisphere split from a singularity defined by a saddle-node bifurcation point (white-filled circles). The evolutions on the back-side hemisphere are π-radial symmetric with those on the front side about the origin $(0,0,0)^{\mathrm{T}}$, and there is also a singularity nexus on the back-side hemisphere located at $(-1,0,0)^{\mathrm{T}}$ with $\Omega_0=-\gamma/(2\kappa)$. In this work, we only study the dynamics on the front-side hemisphere.

SUPPLEMENTARY NOTE 7. ORDER PARAMETER

To better signify the spontaneous chiral symmetry-breaking process behind the "pitchfork" bifurcation in Fig. 2g of the main text, we define the relative population of the two chiral states $|\mathrm{CW}\rangle$ and $|\mathrm{CCW}\rangle$ as the order parameter. To do so, we first expend the state vector in the $\{|\mathrm{CW}\rangle,|\mathrm{CCW}\rangle\}$ basis, $|\psi\rangle=c_{\mathrm{cw}}|\mathrm{CW}\rangle+c_{\mathrm{ccw}}|\mathrm{CCW}\rangle$. The order parameter or the relative population of the two chiral states is defined as

$$\begin{aligned}\mathcal{N}&=(|c_{\mathrm{cw}}|^2-|c_{\mathrm{ccw}}|^2)/(|c_{\mathrm{cw}}|^2+|c_{\mathrm{ccw}}|^2)\\&=\frac{\langle\psi|\mathrm{CW}\rangle\langle\mathrm{CW}|\psi\rangle-\langle\psi|\mathrm{CCW}\rangle\langle\mathrm{CCW}|\psi\rangle}{\langle\psi|\mathrm{CW}\rangle\langle\mathrm{CW}|\psi\rangle+\langle\psi|\mathrm{CCW}\rangle\langle\mathrm{CCW}|\psi\rangle}.\end{aligned}\tag{S.53}$$

Based on the relation (S.52), we can obtain

$$\begin{aligned}c_{\mathrm{cw}}&=\langle\mathrm{CW}|\psi\rangle=\sin(\pi/4+\vartheta/2)\mathrm{e}^{i(\pi/2-\phi)},\\c_{\mathrm{ccw}}&=\langle\mathrm{CCW}|\psi\rangle=\cos(\pi/4+\vartheta/2).\end{aligned}\tag{S.54}$$

Substituting (S.54) into (S.53), we can obtain a more simplified form of the order parameter

$$\mathcal{N}=\sin\phi\sin\vartheta,\tag{S.55}$$

which is exactly the chirality S_2 of the state vector $|\psi\rangle$. The simulated order parameter as a function of rotation velocity Ω and degeneracy condition $\Delta\omega$ is shown in Supplementary Fig. **10**.

SUPPLEMENTARY NOTE 8. HIGH SENSITIVITY AT SINGULARITY NEXUS

A. Sensitivity at singularity nexus

There is a sharp and continuous transition at the singularity nexus (Fig. 4a), which indicates that a small perturbation in the parameters would result in a drastic change of the closed-loop frequency ω_{d}^*. Here,

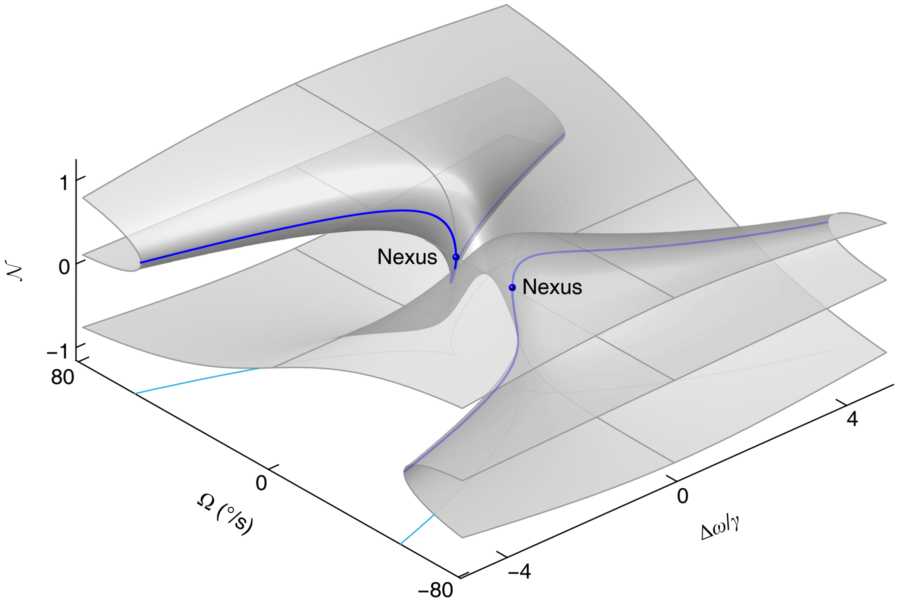

Supplementary Figure 10. **Order parameter.** Calculated order parameter $\mathcal{N}$ as a function of rotation velocity and degeneracy condition, which shows a folded surface as well.

we consider the perturbation ϵ that would affect the natural frequencies of the two modes and thus the degeneracy condition, $\Delta\omega = \alpha\epsilon$, where α is the affecting coefficient. For simplicity, we just assume $\alpha = 1$ in the following analyses.

The phase-tracked frequency at the singularity nexus as a function of $\Delta\omega$ can be obtained by substituting $\Omega_0 = \gamma/(2\kappa)$ into the cubic equation (S.39),

$$\{[\omega_{\rm d}^*(\Omega_0) - \omega_1] - \Delta\omega\}^2[\omega_{\rm d}^*(\Omega_0) - \omega_1] + \frac{\gamma^2}{4}\Delta\omega = 0.$$

It should be noted that natural frequency ω_1 may also be affected by the perturbation. Therefore, we can regard the indirect variable $\delta\omega_{\rm X} \equiv \omega_{\rm d}^*(\Omega_0) - \omega_1$ as the sensing output, which represents the shift of the phase-tracked frequency at the singularity nexus from the driven mode natural frequency, as shown in Fig. 4b. By introducing the perturbation $\Delta\omega = \epsilon$, we get the input-output sensing equation at the singularity nexus,

$$(\delta\omega_{\rm X} - \epsilon)^2\,\delta\omega_{\rm X} + \frac{\gamma^2}{4}\epsilon = 0. \tag{S.56}$$

By solving the above cubic equation about $\delta\omega_{\rm X}$, we can obtain the output $\delta\omega_{\rm X}$ as a function of input ϵ,

$$\begin{aligned}\delta\omega_{\rm X} &\equiv \omega_{\rm d}^*(\Omega_0) - \omega_1 \qquad\qquad (\text{S.57})\\ &= \frac{2}{3}\epsilon + \sqrt[3]{-\epsilon\left(\frac{\gamma^2}{8} + \frac{\epsilon^2}{27}\right) + \frac{\gamma\epsilon}{2}\sqrt{\frac{\gamma^2}{16} + \frac{\epsilon^2}{27}}} + \sqrt[3]{-\epsilon\left(\frac{\gamma^2}{8} + \frac{\epsilon^2}{27}\right) - \frac{\gamma\epsilon}{2}\sqrt{\frac{\gamma^2}{16} + \frac{\epsilon^2}{27}}}.\end{aligned}$$

In the vicinity of the singularity nexus $-0.25\gamma \le \epsilon \le 0.25\gamma$, $\delta\omega_{\mathrm{X}}$ decreases monotonically to ϵ. The absolute input-output data in Fig. 4c of the main text drawn on the logarithmic scale is given by the red solid curve in Fig. 4d of the main text. The slope of 1/3 in the logarithmic plot confirms that perturbation along the $\Delta\omega$ axis around the singularity nexus can provide enhanced output on the order of $\epsilon^{1/3}$. This property makes a good sensor with very high sensitivity near the singularity nexus.

B. Sensitivity comparison with binary exceptional point and diabolic point

We compare the sensitivity of the singularity nexus with that of the binary exceptional point (EP), which is the degeneracy point of the non-Hermitian systems with two degrees of freedom. We consider a binary non-Hermitian system described by the equations of motion

$$\mathrm{i}\begin{bmatrix}\dot{c}_1\\ \dot{c}_2\end{bmatrix}=\begin{bmatrix}\omega_1-\frac{\mathrm{i}}{2}\gamma_1 & \frac{1}{2}g\\ \frac{1}{2}g & \omega_2-\frac{\mathrm{i}}{2}\gamma_2\end{bmatrix}\begin{bmatrix}c_1\\ c_2\end{bmatrix}, \tag{S.58}$$

where $c_{1,2}$ are the bosonic operators of the two coupled modes, and g is the strength of the coherent coupling. The two modes have different damping rates, $\gamma_1 \neq \gamma_2$. Here, we consider a perturbation that can affect the degeneracy condition $\epsilon = \Delta\omega = \omega_2 - \omega_1$, just like the case of the singularity nexus. The Hamiltonian matrix with perturbation can be rewritten as

$$\begin{bmatrix}\omega_0-\epsilon/2-\frac{\mathrm{i}}{2}\gamma_1 & \frac{1}{2}g\\ \frac{1}{2}g & \omega_0+\epsilon/2-\frac{\mathrm{i}}{2}\gamma_2\end{bmatrix}=(\omega_0-\frac{\mathrm{i}}{2}\gamma_0)\mathbf{I}+\frac{1}{2}\begin{bmatrix}-\epsilon+\frac{\mathrm{i}}{2}\Delta\gamma & g\\ g & \epsilon-\frac{\mathrm{i}}{2}\Delta\gamma\end{bmatrix}, \tag{S.59}$$

where $\mathbf{I}$ indicates the identity matrix, $\omega_0 = (\omega_1 + \omega_2)/2$, $\gamma_0 = (\gamma_1 + \gamma_2)/2$, and $\Delta\gamma = \gamma_2 - \gamma_1$. The first term on the right side of (S.59) is the global phase describing the overall decaying behaviour. The second term on the right side of (S.59) describes a parity-time-symmetric system. The eigenvalues of the overall system are given by

$$\lambda_{\pm}=(\omega_0-\frac{\mathrm{i}}{2}\gamma_0)\pm\frac{1}{2}\sqrt{\left(\epsilon-\frac{\mathrm{i}}{2}\Delta\gamma\right)^2+g^2}$$

If the system operates near the exceptional point, $g = \Delta\gamma/2$, the eigenvalues as functions of perturbation are further given by

$$\lambda_{\pm}^{\mathrm{EP}}=(\omega_0-\frac{\mathrm{i}}{2}\gamma_0)\pm\frac{1}{2}\sqrt{\epsilon^2-\mathrm{i}\epsilon\Delta\gamma}. \tag{S.60}$$

The real (imaginary) parts of the eigenvalues indicate the eigenfrequencies (damping rates) of the two modes. The eigenfrequency split as a function of perturbation is further given by

$$\delta\omega_{\mathrm{EP}}=\Re[\lambda_{+}^{\mathrm{EP}}]-\Re[\lambda_{-}^{\mathrm{EP}}]=\sqrt{\frac{\epsilon^2+|\epsilon|\sqrt{\epsilon^2+\Delta\gamma^2}}{2}}, \tag{S.61}$$

which acts as the sensing output of the exceptional-point system, and is drawn in the vicinity of EP by the blue dashed curve in Fig. 4c of the main text. In this calculation, the damping difference of the EP system is chosen to be equal to the dissipation rate of the phase-tracked cubic singularity system, $\Delta\gamma = \gamma$. The corresponding logarithmic scale plot of $\delta\omega_{\mathrm{EP}}$ is given by the blue dashed curve in Fig. 4d of the main text. The slope of 1/2 in the logarithmic plot confirms that the perturbation along the $\Delta\omega$ axis around the exceptional point can provide output on the order of $\epsilon^{1/2}$. It is noteworthy that the sensitivity near the singularity nexus is larger than the sensitivity near EP.

For better comparison, the sensitivity around the diabolic point (DP) is also calculated. We consider a binary system described by the equations of motion

$$\mathrm{i}\begin{bmatrix}\dot{c}_1\\ \dot{c}_2\end{bmatrix} = \begin{bmatrix}\omega_1 - \frac{\mathrm{i}}{2}\gamma & \frac{1}{2}g\\ \frac{1}{2}g & \omega_2 - \frac{\mathrm{i}}{2}\gamma\end{bmatrix}\begin{bmatrix}c_1\\ c_2\end{bmatrix}. \tag{S.62}$$

We consider a perturbation that can affect the degeneracy condition $\epsilon = \Delta\omega = \omega_2 - \omega_1$, as well. The Hamiltonian matrix with perturbation is rewritten as

$$\begin{bmatrix}\omega_0 - \epsilon/2 - \frac{\mathrm{i}}{2}\gamma & \frac{1}{2}g\\ \frac{1}{2}g & \omega_0 + \epsilon/2 - \frac{\mathrm{i}}{2}\gamma\end{bmatrix} = (\omega_0 - \frac{\mathrm{i}}{2}\gamma)\mathbf{I} + \frac{1}{2}\begin{bmatrix}-\epsilon & g\\ g & \epsilon\end{bmatrix}, \tag{S.63}$$

where $\omega_0 = (\omega_1 + \omega_2)/2$. The eigenvalues of the overall system are given by

$$\lambda_{\pm}^{\mathrm{DP}} = (\omega_0 - \frac{\mathrm{i}}{2}\gamma) \pm \frac{1}{2}\sqrt{\epsilon^2 + g^2}.$$

If the system operates near the diabolic point, $g = 0$, the eigenfrequencies as functions of the perturbation are further given by

$$\omega_{\pm}^{\mathrm{DP}} = \omega_0 \pm \frac{1}{2}|\epsilon|. \tag{S.64}$$

The eigenfrequency split as a function of the perturbation is further given by

$$\delta\omega_{\mathrm{DP}} = \omega_{+}^{\mathrm{DP}} - \omega_{-}^{\mathrm{DP}} = |\epsilon|, \tag{S.65}$$

which is the sensing output of the conventional DP system, and is drawn in the vicinity of the DP by the black dot-dashed curve in Fig. 4c of the main text. The slope of the corresponding logarithmic scale plot of $\delta\omega_{\mathrm{DP}}$ is one, as shown by the black dot-dashed curve in Fig. 4d of the main text. Both nexus and EP show sensitivity enhancement compared with DP.

SUPPLEMENTARY DISCUSSION 1. VOLTAGE-CONTROLLED NONRECIPROCAL STATE TRANSFER

A. Concept of the nonreciprocal state transfer

Previous studies have demonstrated the achievement of nonreciprocal state transfer by dynamically encircling the second-order EP singularity [12–17]. More recently, it has been shown that nonreciprocal state transfer can also be achieved by encircling the cubic EP singularity [18, 19]. Furthermore, the necessity of the winding process for nonreciprocity has been challenged, as it has been discovered that nonreciprocal state transfer can be realized by traversing an EP-excluding cycle [20], leveraging the non-trivial landscape of the spectrum surface near the EP. In this study, we present the capability of the PhT singularity to enable closed-loop controlled nonreciprocal state transfer. Additionally, compared to previous approaches that rely on two-parameter-controlled encircling, we demonstrate that nonreciprocal state transfer can be achieved using a single-parameter-controlled traversal.

We now consider a 1D closed loop in the parameter plane, where the tuning voltage V_t is continuously varied while the rotation velocity Ω remains a constant value that is larger than Ω_0. The loop starts and ends in the bistable region and intersects both projected singularity loci. At the start/end point, there may exist two bistable PhT states, with the high-frequency state denoted as $|a\rangle$ and the low-frequency state denoted as $|b\rangle$. When traversing the loop in the down-up-down (up-down-up) direction, as shown in Supplementary Fig. **11**a (b), irrespective of the initial bistable state, the system always ends at $|b\rangle$ ($|a\rangle$), as demonstrated in Supplementary Fig. **11**c and d (e and f). The ending location only relies on the direction of the parametric trajectory and is independent of the starting location.

Here, we show that by steering the tuning voltage V_t along the 1D closed parametric trajectories in Supplementary Fig. **11**a,b, we can realize a nonreciprocal state transfer. The voltage-controlled trajectories start/end at the voltage of $V_t = -18.55$ V, referred to as PhT states $|a\rangle$ and $|b\rangle$ on the upper and lower stable branch with PhT frequency of 3852.155 Hz and 3851.877 Hz, respectively.

We consider the closed trajectory defined by V_t values of -18.55 V $\to -20$ V $\to -17$ V $\to -18.55$ V and labelled by '$\downarrow$' in Supplementary Fig. **11**a. The '$\downarrow$' traversing process is described by a transforming matrix $\mathbf{U}_\downarrow$, which can transfer state $|a\rangle$ to state $|b\rangle$, $\mathbf{U}_\downarrow \begin{bmatrix} |a| \\ 0 \end{bmatrix} = \begin{bmatrix} 0 \\ |b| \end{bmatrix}$, but it cannot transfer state $|b\rangle$ to state $|a\rangle$, $\mathbf{U}_\downarrow \begin{bmatrix} 0 \\ |b| \end{bmatrix} = \begin{bmatrix} 0 \\ |b| \end{bmatrix}$. Here $|a|$ and $|b|$ represent the real amplitudes of PhT states $|a\rangle$ and $|b\rangle$

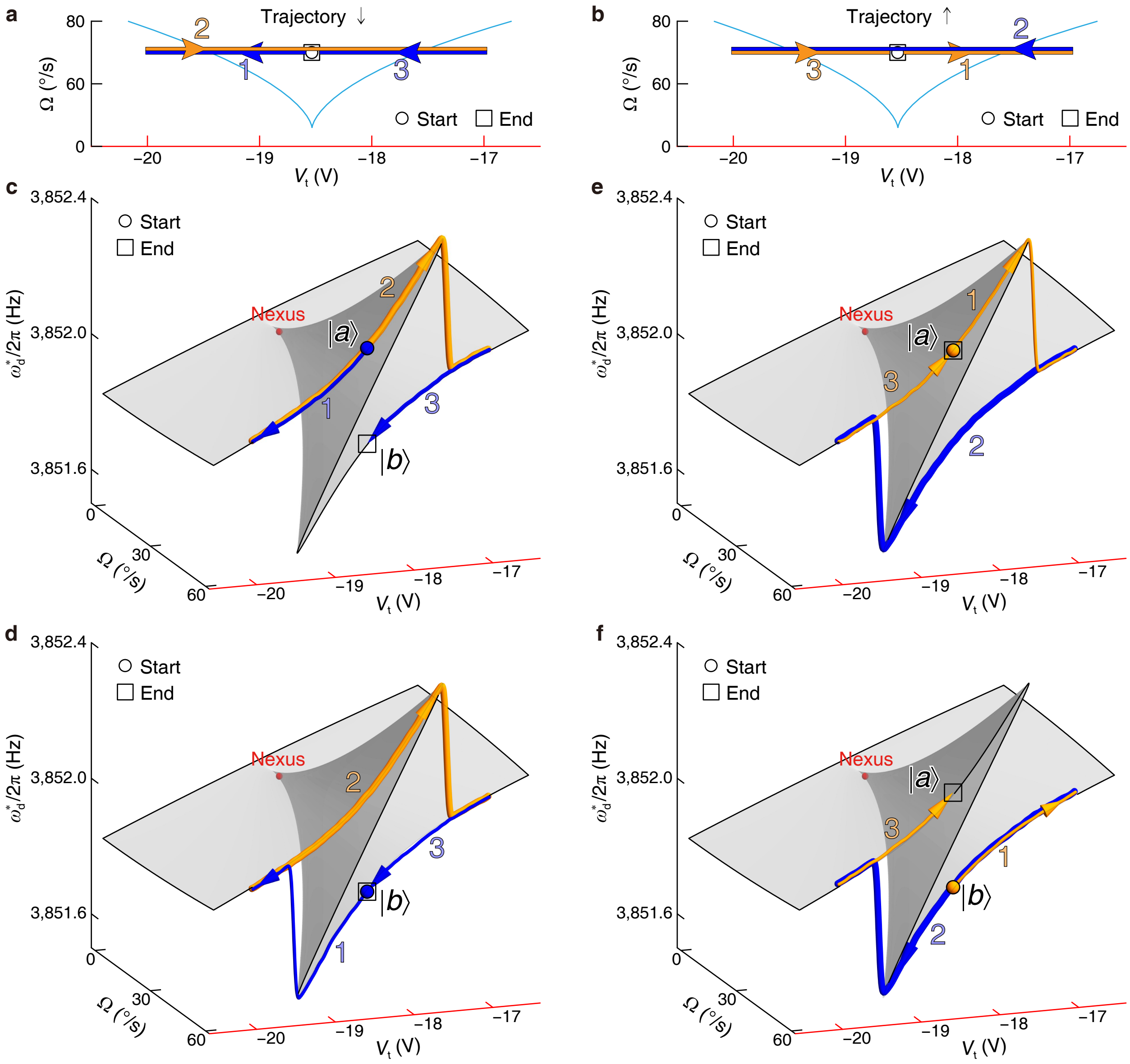

Supplementary Figure 11. **Voltage-controlled nonreciprocal state transfer. a** The 1D closed trajectory ↓ defined by V_t values of -18.55 V $\rightarrow$ -20 V $\rightarrow$ -17 V $\rightarrow$ -18.55 V and $\Omega = 60°/\mathrm{s}$. This trajectory starts/ends at bistable region and crosses both projected singularity loci (cyan curves). The trajectory fragments with increasing or decreasing V_t are depicted in orange or blue colours. The outlined numbers indicate the order of traversal. **b** The closed trajectory ↑ that is the reverse of trajectory ↓. **c,d** Adiabatic evolutions starting at (**c**) $|a\rangle$ located at the high-frequency bistable branch or (**d**) $|b\rangle$ located at the low-frequency bistable branch following the ↓ trajectory process. The ↓ traversal process facilitates the transfer from $|a\rangle$ to $|b\rangle$, yet it cannot effectuate the reverse transfer from $|b\rangle$ to $|a\rangle$. In other words, the ↓ trajectory-enabled state transfer from $|a\rangle$ to $|b\rangle$ is nonreciprocal. **e,f** Same as **c,d**, respectively, for the reversed ↑ trajectory process. The state transfer from $|b\rangle$ to $|a\rangle$ in ↑ traversing process is also nonreciprocal. The ending location is only related to the traversing direction.

respectively. We thereby obtain an asymmetric transforming matrix,

$$\mathbf{U}_{\downarrow} = \begin{bmatrix} 0 & 0 \\ \frac{|b|}{|a|} & 1 \end{bmatrix},$$

representing a ideal nonreciprocal state transfer.

Similarly, the reversed closed '↑' trajectory defined by V_{t} values of −18.55 V → −17 V → −20 V → −18.55 V in Supplementary Fig. **11**b is described by a transforming matrix $\mathbf{U}_{\uparrow}$, which can transfer state $|b\rangle$ to state $|a\rangle$, $\mathbf{U}_{\uparrow}\begin{bmatrix} 0 \\ |b| \end{bmatrix} = \begin{bmatrix} |a| \\ 0 \end{bmatrix}$, but it cannot transfer state $|a\rangle$ to state $|b\rangle$, $\mathbf{U}_{\uparrow}\begin{bmatrix} |a| \\ 0 \end{bmatrix} = \begin{bmatrix} |a| \\ 0 \end{bmatrix}$. Therefore $\mathbf{U}_{\uparrow}$ is also an asymmetric transforming matrix representing an ideal nonreciprocal state transfer,

$$\mathbf{U}_{\uparrow} = \begin{bmatrix} 1 & \frac{|a|}{|b|} \\ 0 & 0 \end{bmatrix}.$$

B. Experimental demonstration of the nonreciprocal state transfer

In practice, the information of the starting and ending points at states $|a\rangle$ and $|b\rangle$ of the four state-transferring processes in Supplementary Fig. **11**c-f are fully measured, which are summarized in Supplementary Table **1**. The states are expressed in a form of $|q_1||1\rangle + |q_2|e^{i\vartheta}|2\rangle$, where the overall phase is gauged away. Here, $|q_1|$ and $|q_2|$ are the amplitudes of the projections of the starting/ending states to the $|1\rangle$ and $|2\rangle$ states, respectively, and ϑ is the relative phase of the two projections. The $|q_1|$, $|q_2|$ have unit of volt, and ϑ have unit of degree. By transforming from the $\{|1\rangle, |2\rangle\}$ to the $\{|a\rangle, |b\rangle\}$ basis, we can extrapolate the amplitudes of the starting and ending states.

For the ↓ process in Supplementary Fig. **11**c, the system is initiated at state $|a_{\downarrow}\rangle$, the amplitude of which is calculated to be $|a_{\downarrow}| = \sqrt{\langle a_{\downarrow}|a_{\downarrow}\rangle} = 73.52$ mV. At the starting point, the state $|b_{\downarrow}\rangle$ is not excited at all, which implies a zero amplitude at state $|b_{\downarrow}\rangle$ at the start. Thereby the amplitude vector at the starting point is $(73.52\ \mathrm{mV}, 0)^{\mathrm{T}}$.

The ↓ traversing process will transfer the starting state $|a_{\downarrow}\rangle$ to the ending state $|b_{\downarrow}\rangle$, the amplitude of which is calculated to be $|b_{\downarrow}| = \sqrt{\langle b_{\downarrow}|b_{\downarrow}\rangle} = 83.72$ mV. At the ending point, state $|a_{\downarrow}\rangle$ is not actuated, so its amplitude is zero at the ending point. The amplitude vector at the ending point is $(0, 83.72\ \mathrm{mV})^{\mathrm{T}}$. So the traversing process in Supplementary Fig. **11**c is described by

$$\mathbf{U}_{\downarrow}\begin{bmatrix} 73.52\ \mathrm{mV} \\ 0 \end{bmatrix} = \begin{bmatrix} 0 \\ 83.72\ \mathrm{mV} \end{bmatrix} \tag{S.66}$$

Supplementary Table 1. The experimentally measured state information for the start and end points.

Process	Measured state information
Suppl. Fig.11c 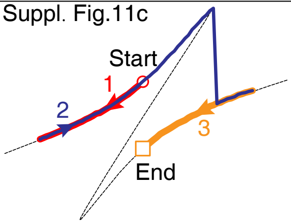	Start state $\lvert a_{\downarrow}\rangle = 48.71\ \text{mV}\ \lvert 1\rangle + 55.07\ \text{mV}\cdot \text{e}^{-\text{i}76.12^{\circ}}\lvert 2\rangle$
	End state $\lvert b_{\downarrow}\rangle = 62.51\ \text{mV}\ \lvert 1\rangle + 55.69\ \text{mV}\cdot \text{e}^{\text{i}76.24^{\circ}}\lvert 2\rangle$
Suppl. Fig.11d 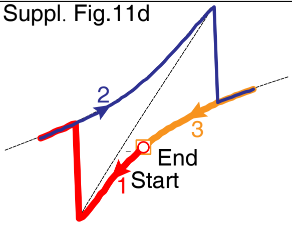	Start state $\lvert b'_{\downarrow}\rangle = 59.02\ \text{mV}\ \lvert 1\rangle + 55.68\ \text{mV}\cdot \text{e}^{\text{i}76.50^{\circ}}\lvert 2\rangle$
	End state $\lvert b''_{\downarrow}\rangle = 63.64\ \text{mV}\ \lvert 1\rangle + 55.16\ \text{mV}\cdot \text{e}^{\text{i}77.33^{\circ}}\lvert 2\rangle$
Suppl. Fig.11e 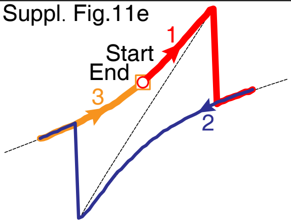	Start state $\lvert a'_{\uparrow}\rangle = 51.04\ \text{mV}\ \lvert 1\rangle + 55.69\ \text{mV}\cdot \text{e}^{-\text{i}75.77^{\circ}}\lvert 2\rangle$
	End state $\lvert a''_{\uparrow}\rangle = 50.77\ \text{mV}\ \lvert 1\rangle + 56.70\ \text{mV}\cdot \text{e}^{-\text{i}75.28^{\circ}}\lvert 2\rangle$
Suppl. Fig.11f 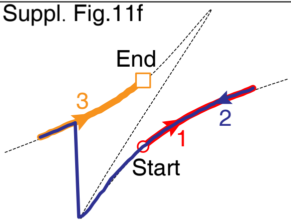	Start state $\lvert b_{\uparrow}\rangle = 63.35\ \text{mV}\ \lvert 1\rangle + 53.25\ \text{mV}\cdot \text{e}^{\text{i}76.66^{\circ}}\lvert 2\rangle$
	End state $\lvert a_{\uparrow}\rangle = 53.00\ \text{mV}\ \lvert 1\rangle + 58.56\ \text{mV}\cdot \text{e}^{-\text{i}76.57^{\circ}}\lvert 2\rangle$

For the $\downarrow$ process in Supplementary Fig. **11**d, the system is initiated at state $\lvert b'_{\downarrow}\rangle$. It is noteworthy that $\lvert b'_{\downarrow}\rangle$ might be slightly different from the previous $\lvert b_{\downarrow}\rangle$ state. We must use the basis $\{\lvert a_{\downarrow}\rangle, \lvert b_{\downarrow}\rangle\}$ when calculating the amplitudes. The amplitude at the $\lvert b_{\downarrow}\rangle$ state is given by $\lvert b_{\downarrow}\rvert = \frac{\sqrt{\langle b_{\downarrow}\vert b'_{\downarrow}\rangle\langle b'_{\downarrow}\vert b_{\downarrow}\rangle}}{\sqrt{\langle b_{\downarrow}\vert b_{\downarrow}\rangle}} = 81.11$ mV. Because of the difference between $\lvert b_{\downarrow}\rangle$ and $\lvert b'_{\downarrow}\rangle$, there may be residual projected amplitude on state $\lvert a_{\downarrow}\rangle$ at the starting point, which is given by $\lvert a_{\downarrow}\rvert = \lvert \frac{\sqrt{\langle a_{\downarrow}\vert b'_{\downarrow}\rangle\langle b'_{\downarrow}\vert a_{\downarrow}\rangle}}{\sqrt{\langle a_{\downarrow}\vert a_{\downarrow}\rangle}} - \frac{\sqrt{\langle a_{\downarrow}\vert b_{\downarrow}\rangle\langle b_{\downarrow}\vert a_{\downarrow}\rangle}}{\sqrt{\langle a_{\downarrow}\vert a_{\downarrow}\rangle}}\rvert = 0.57$ mV. The $\frac{\sqrt{\langle a_{\downarrow}\vert b'_{\downarrow}\rangle\langle b'_{\downarrow}\vert a_{\downarrow}\rangle}}{\sqrt{\langle a_{\downarrow}\vert a_{\downarrow}\rangle}}$ term indicates the projection of $\lvert b'_{\downarrow}\rvert$ to $\lvert a_{\downarrow}\rangle$. Because the $\{\lvert a_{\downarrow}\rangle, \lvert b_{\downarrow}\rangle\}$ basis may not be orthogonal, this projection should be compensated by the nonorthogonality projection of the basis itself $\frac{\sqrt{\langle a_{\downarrow}\vert b_{\downarrow}\rangle\langle b_{\downarrow}\vert a_{\downarrow}\rangle}}{\sqrt{\langle a_{\downarrow}\vert a_{\downarrow}\rangle}}$. Thus, the amplitude vector at the starting point is $(0.57\ \text{mV}, 81.11\ \text{mV})^{\text{T}}$.

The $\downarrow$ traversing process will transfer the starting state $\lvert b'_{\downarrow}\rangle$ to the ending state $\lvert b''_{\downarrow}\rangle$. At the ending point, The amplitude at the $\lvert b_{\downarrow}\rangle$ mode is given by $\lvert b_{\downarrow}\rvert = \frac{\sqrt{\langle b_{\downarrow}\vert b''_{\downarrow}\rangle\langle b''_{\downarrow}\vert b_{\downarrow}\rangle}}{\sqrt{\langle b_{\downarrow}\vert b_{\downarrow}\rangle}} = 84.21$ mV. The difference between $\lvert b_{\downarrow}\rangle$ and $\lvert b''_{\downarrow}\rangle$ may also introduce residual projected amplitude on state $\lvert a_{\downarrow}\rangle$ at the ending point, $\lvert a_{\downarrow}\rvert = \lvert \frac{\sqrt{\langle a_{\downarrow}\vert b''_{\downarrow}\rangle\langle b''_{\downarrow}\vert a_{\downarrow}\rangle}}{\sqrt{\langle a_{\downarrow}\vert a_{\downarrow}\rangle}} - \frac{\sqrt{\langle a_{\downarrow}\vert b_{\downarrow}\rangle\langle b_{\downarrow}\vert a_{\downarrow}\rangle}}{\sqrt{\langle a_{\downarrow}\vert a_{\downarrow}\rangle}}\rvert = 0.67$ mV. Therefore, the amplitude vector at the ending point

is $(0.67\ \text{mV}, 84.21\ \text{mV})^{\mathrm{T}}$. To sum up, the traversing process in Supplementary Fig. **11**d is described by

$$\mathbf{U}_{\downarrow}\begin{bmatrix} 0.57\ \text{mV} \\ 81.11\ \text{mV} \end{bmatrix} = \begin{bmatrix} 0.67\ \text{mV} \\ 84.21\ \text{mV} \end{bmatrix} \tag{S.67}$$

Combining (S.66) and (S.67), we get the transforming matrix of the $\downarrow$ traversing process in our experiment,

$$\mathbf{U}_{\downarrow} = \begin{bmatrix} 0 & 8.26\times 10^{-3} \\ 1.14 & 1.03 \end{bmatrix}.$$

The isolation ratio $\mathbf{U}_{\downarrow}(1,2)/\mathbf{U}_{\downarrow}(2,1)$ of the $\downarrow$ nonreciprocal state transfer is calculated to be -43 dB.

Then we consider the $\uparrow$ in Supplementary Fig. **11**f, the system is initiated at state $|b_{\uparrow}\rangle$, the amplitude of which is calculated to be $|b_{\uparrow}| = \sqrt{\langle b_{\uparrow}|b_{\uparrow}\rangle} = 82.76$ mV. At the starting point, the amplitude at $|a_{\uparrow}\rangle$ is zero. Thereby the amplitude vector at the starting point is $(0, 82.76\ \text{mV})^{\mathrm{T}}$.

The $\uparrow$ traversing process will transfer the starting state $|b_{\uparrow}\rangle$ to the ending state $|a_{\uparrow}\rangle$, the amplitude of which is calculated to be $|a_{\uparrow}| = \sqrt{\langle a_{\uparrow}|a_{\uparrow}\rangle} = 78.98$ mV. At the ending point, the amplitude at $|b_{\uparrow}\rangle$ is zero. The amplitude vector at the ending point is $(78.98\ \text{mV}, 0)^{\mathrm{T}}$. So the traversing process in Supplementary Fig. **11**f is described by

$$\mathbf{U}_{\uparrow}\begin{bmatrix} 0 \\ 82.76\ \text{mV} \end{bmatrix} = \begin{bmatrix} 78.98\ \text{mV} \\ 0 \end{bmatrix} \tag{S.68}$$

For the $\uparrow$ process in Supplementary Fig. **11**e, the system is initiated at state $|a'_{\uparrow}\rangle$. We use the basis $\{|a_{\uparrow}\rangle, |b_{\uparrow}\rangle\}$ when calculating the amplitudes. At the starting point, the $|a_{\uparrow}\rangle$ state amplitude is given by $|a_{\uparrow}| = \frac{\sqrt{\langle a_{\uparrow}|a'_{\uparrow}\rangle a'_{\uparrow}|a_{\uparrow}\rangle}}{\sqrt{\langle a_{\uparrow}|a_{\uparrow}\rangle}} = 75.54$ mV. The difference between $|a'_{\uparrow}\rangle$ and $|a_{\uparrow}\rangle$ may introduce residual projected amplitude on state $|b_{\uparrow}\rangle$ at the starting point, $|b_{\uparrow}| = |\frac{\sqrt{\langle b_{\uparrow}|a'_{\uparrow}\rangle\langle a'_{\uparrow}|b_{\uparrow}\rangle}}{\sqrt{\langle b_{\uparrow}|b_{\uparrow}\rangle}} - |\frac{\sqrt{\langle b_{\uparrow}|a_{\uparrow}\rangle\langle a_{\uparrow}|b_{\uparrow}\rangle}}{\sqrt{\langle b_{\uparrow}|b_{\uparrow}\rangle}}| = 0.21$ mV. Thus, the amplitude vector at the starting point is $(75.54\ \text{mV}, 0.21\ \text{mV})^{\mathrm{T}}$.

The $\uparrow$ traversing process will transfer the starting state $|a'_{\uparrow}\rangle$ to the ending state $|a''_{\uparrow}\rangle$. At the ending point, The amplitude at the $|a_{\uparrow}\rangle$ mode is given by $|a_{\uparrow}| = \frac{\sqrt{\langle a_{\uparrow}|a''_{\uparrow}\rangle\langle a''_{\uparrow}|a_{\uparrow}\rangle}}{\sqrt{\langle a_{\uparrow}|a_{\uparrow}\rangle}} = 76.10$ mV. The difference between $|ta''_{\uparrow}\rangle$ and $|a_{\uparrow}\rangle$ may also introduce residual projected amplitude on state $|b_{\uparrow}\rangle$ at the ending point, $|b_{\uparrow}| = |\frac{\sqrt{\langle b_{\uparrow}|a''_{\uparrow}\rangle\langle a''_{\uparrow}|b_{\uparrow}\rangle}}{\sqrt{\langle b_{\uparrow}|b_{\uparrow}\rangle}} - |\frac{\sqrt{\langle b_{\uparrow}|a_{\uparrow}\rangle\langle a_{\uparrow}|b_{\uparrow}\rangle}}{\sqrt{\langle b_{\uparrow}|b_{\uparrow}\rangle}}| = 0.08$ mV. Therefore, the amplitude vector at the ending point is $(76.10\ \text{mV}, 0.08\ \text{mV})^{\mathrm{T}}$. To sum up, the traversing process in Supplementary Fig. **11**e is described by

$$\mathbf{U}_{\uparrow}\begin{bmatrix} 75.54\ \text{mV} \\ 0.21\ \text{mV} \end{bmatrix} = \begin{bmatrix} 76.10\ \text{mV} \\ 0.08\ \text{mV} \end{bmatrix} \tag{S.69}$$

Combining (S.68) and (S.69), we get the transforming matrix of the $\uparrow$ traversing process in our experiment,

$$\mathbf{U}_{\uparrow} = \begin{bmatrix} 1.00 & 0.95 \\ 1.06\times 10^{-3} & 0 \end{bmatrix}.$$

The isolation ratio $\mathbf{U}_{\uparrow}(1,2)/\mathbf{U}_{\uparrow}(2,1)$ of the $\uparrow$ nonreciprocal state transfer is calculated to be 59 dB.

In this study, nonreciprocal state transfer is achieved solely by manipulating the tuning voltage V_{t}, while keeping the angular velocity Ω at a constant value. It is worth noting that the Ω value of the V_{t}-controlled nonreciprocal process can be changed almost arbitrarily, as long as the starting/ending points reside within the bistable region and both projected singularity loci are crossed. Leveraging the electrostatic tunability of our device, the PhT cubic singularity has facilitated the realization of *voltage-controlled nonreciprocity*, offering a desirable feature for practical applications.

SUPPLEMENTARY DISCUSSION 2. PHASE-TRACKED CUBIC SINGULARITIES IN LINEAR COHERENT COUPLING

In the main text, we demonstrate the cubic singularity in the phase-tracked oscillations of a Coriolis-coupled microelectromechanical resonator. Here, we show theoretically that this kind of cubic singularity may also ubiquitously reside in binary systems coherently coupled by an ordinary linear stiffness mechanism, as shown in Supplementary Fig. **12**. Oscillator 1 is actuated by an a.c. force $F = F_0\cos(\omega_{\mathrm{d}}t)$, whereas the other one is left free. The Hamiltonian of this driven coupled system is given by

$$H_{\mathrm{L}} = \hbar\omega_1 b_1^* b_1 + \hbar\omega_2 b_2^* b_2 + \frac{1}{2}\hbar g\left(b_1^* b_2 + b_1 b_2^*\right) - \hbar f_0 (b_1 \mathrm{e}^{\mathrm{i}\omega_{\mathrm{d}}t} + b_1^* \mathrm{e}^{-\mathrm{i}\omega_{\mathrm{d}}t}), \tag{S.70}$$

where g is the strength of the linear coupling, and f_0 is the normalized force defined by $\frac{1}{2}\frac{F_0}{\sqrt{2\hbar m\omega_1}}$. The eigenfrequencies of the linear-coupling system are

$$\omega'_{\pm} = \frac{\omega_1+\omega_2}{2} \pm \frac{1}{2}\sqrt{\Delta\omega^2 + g^2}. \tag{S.71}$$

The Langevin equation of motion about the creation operator b_j^* $(j = 1, 2)$ is further given by

$$\frac{\mathrm{d}}{\mathrm{d}t}b_j^* = \frac{\mathrm{i}}{\hbar}\{H_{\mathrm{L}}, b_j^*\} - \frac{\gamma}{2}b_j^* + \xi_j^*. \tag{S.72}$$

where γ is the dissipation rate of the degenerate modes and ξ_j^* indicates the thermal Langevin force acting on mode b_j^*. The equations of motion can be obtained,

$$\begin{bmatrix} \dot{b}_1^* \\ \dot{b}_2^* \end{bmatrix} = \begin{bmatrix} \mathrm{i}\omega_1 - \frac{1}{2}\gamma & \frac{\mathrm{i}}{2}g \\ \frac{\mathrm{i}}{2}g & \mathrm{i}\omega_2 - \frac{1}{2}\gamma \end{bmatrix} \begin{bmatrix} b_1^* \\ b_2^* \end{bmatrix} - \begin{bmatrix} \mathrm{i}f_0 \\ 0 \end{bmatrix} \mathrm{e}^{\mathrm{i}\omega_{\mathrm{d}}t}. \tag{S.73}$$

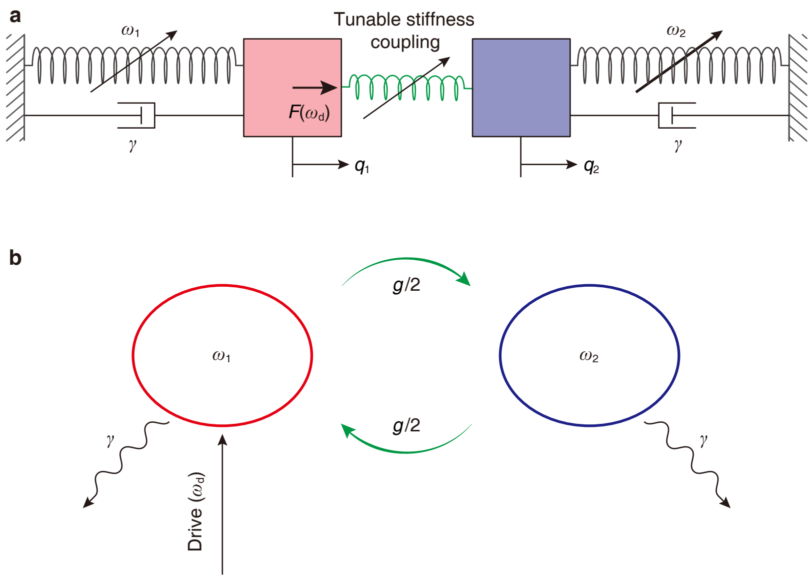

Supplementary Figure 12. **Schematic of the linear stiffness coupling.** (**a**) Two oscillators (modes) with adjustable natural frequencies (stiffness) coupled by a tunable spring. (**b**) Conceptual drawing of the linear coupling. The two oscillators have identical dissipation rates. Mode 1 is actuated alone.

Here, the fluctuation terms are neglected.

The natural frequency difference of the coupled modes and the stiffness coupling strength can be adjusted in real-time. We then transform to a frame rotating at the driving frequency ω_d by writing $b^*_{1,2} = \beta_{1,2}e^{i\omega_d t}$. The equations of motion about the complex amplitudes $\alpha_{1,2}$ in the rotating-frame are given by

$$\begin{bmatrix} \dot{\beta}_1 \\ \dot{\beta}_2 \end{bmatrix} = \begin{bmatrix} -i\Delta_1 - \frac{1}{2}\gamma & \frac{i}{2}g \\ \frac{i}{2}g & -i\Delta_2 - \frac{1}{2}\gamma \end{bmatrix} \begin{bmatrix} \beta_1 \\ \beta_2 \end{bmatrix} - \begin{bmatrix} i f_0 \\ 0 \end{bmatrix},$$

where $\Delta_{1,2} \equiv \omega_d - \omega_{1,2}$ denote the driving detunings. By considering the steady-state condition $\dot{\beta}_{1,2} = 0$, we have $\beta_{1,2} = f_0 \chi'_{1,2}(\omega_d)$, where the mechanical susceptibilities $\chi'_{1,2}$ of the linear coupling case are given by

$$\begin{aligned} \chi'_1(\omega_d) &= \frac{\omega_d - \omega_2 - i\gamma/2}{[i(\omega_d - \omega_1) + \gamma/2]\,[i(\omega_d - \omega_2) + \gamma/2] + g^2/4} \\ &= \frac{\omega_2 - \omega_d + i\gamma/2}{(\omega_d - \omega'_+ - i\gamma/2)(\omega_d - \omega'_- - i\gamma/2)}, \\ \chi'_2(\omega_d) &= \frac{g/2}{[i(\omega_d - \omega_1) + \gamma/2]\,[i(\omega_d - \omega_2) + \gamma/2] + g^2/4} \\ &= \frac{-g/2}{(\omega_d - \omega'_+ - i\gamma/2)(\omega_d - \omega_- - i\gamma/2)}. \end{aligned}$$

Based on the relations $q_{1,2} = \left(b_{1,2} + b^*_{1,2}\right)/(\sqrt{2m\omega_{1,2}})$, the frequency responses of the amplitudes and

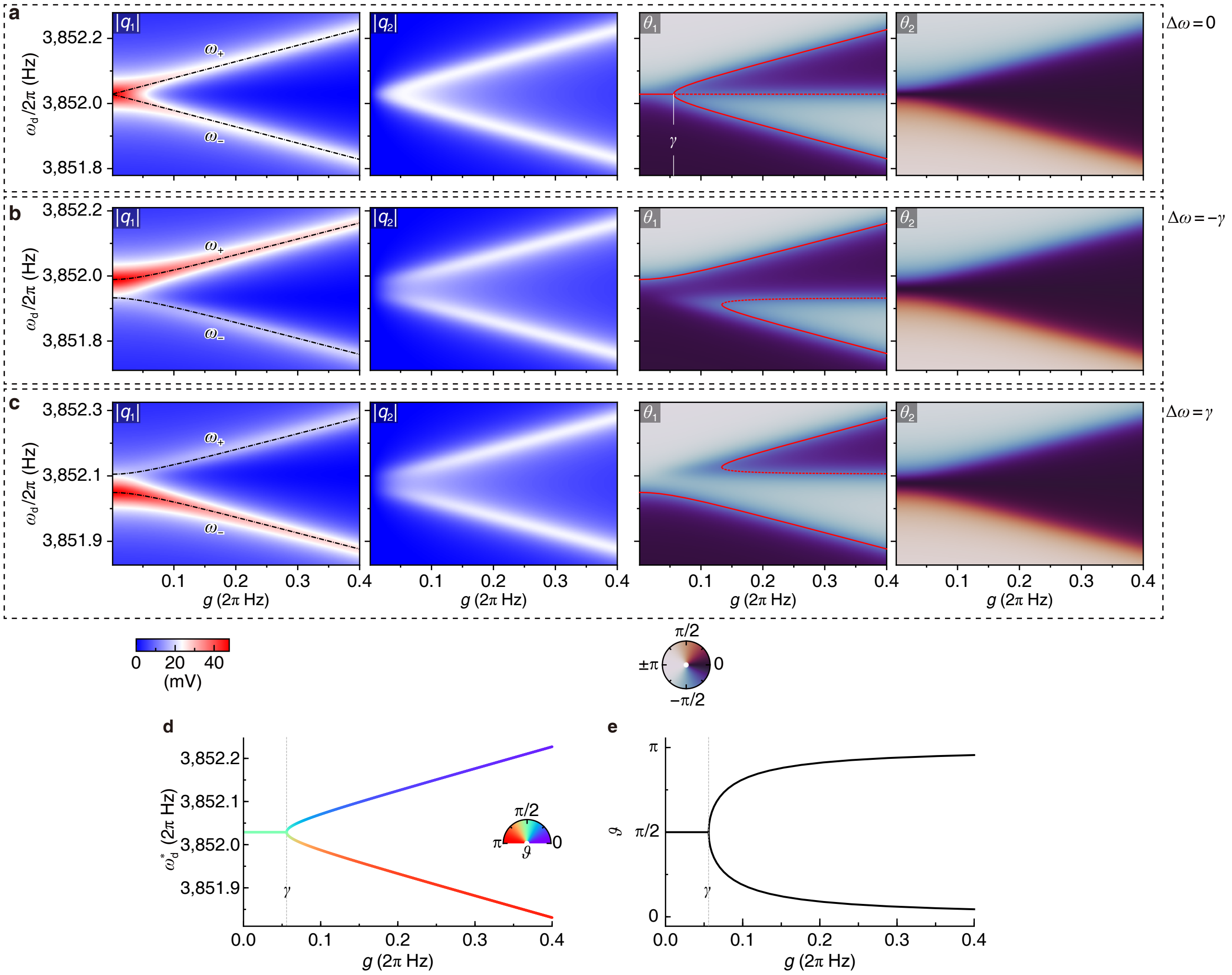

Supplementary Figure 13. Simulated frequency responses of the linear-coupling system and the symmetry breaking in the phase-tracked oscillations. (**a** to **c**) Frequency responses of the amplitudes $|q_{1,2}|(\omega_\mathrm{d})$ and phases $\theta_{1,2}(\omega_\mathrm{d})$ as functions of the linear-coupling strength and natural-frequency difference $\Delta\omega$. (**d**,**e**) Symmetry breaking in the phase-tracked oscillations. In the mode-matched condition, $\Delta\omega = 0$, the driven-mode phase θ_1 is locked to $-\pi/2$. The phase-tracked frequency ω_d^* (d) and relative phase $\vartheta = \theta_2 - \theta_1$ (e) as functions of the linear-coupling strength g showing a symmetry-breaking process at the critical rate $g = \gamma$.

phases of the linear-coupling system are given by

$$|q_1| = |\chi_1'|\frac{F_0}{2m\omega_1}, \tag{S.74}$$

$$|q_2| = |\chi_2'|\frac{F_0}{2m\sqrt{\omega_1\omega_2}}, \tag{S.75}$$

$$\theta_1 = \angle\chi_1', \tag{S.76}$$

$$\theta_2 = \angle\chi_2'. \tag{S.77}$$

The simulated frequency responses as functions of the natural-frequency difference $\Delta\omega = \omega_2 - \omega_1$ and coupling strength g are shown in Supplementary Fig. **13**A to C. The dynamical parameters of the system used here are identical to those of the main text.

It is noteworthy that the amplitude-frequency responses $|q_{1,2}|(\omega_\mathrm{d})$ of both modes and the phase-frequency response of mode 1 $\theta_1(\omega_\mathrm{d})$ of the linear coupling system in Supplementary Fig. **13**a to c are identical to those of the Coriolis coupling system (Supplementary Fig. **4** and Supplementary Fig. **5**). However, the phase frequency responses $\theta_2(\omega_\mathrm{d})$ of mode 2 of the linear coupling system in Supplementary Fig. **13**a to c are different to those of the Coriolis coupling system (Supplementary Fig. **4** and Supplementary Fig. **5**).

If we track the driven-mode phase θ_1 to $-\pi/2$, the phase-tracked closed-loop frequency ω_d^* is derived to be

$$(\omega_\mathrm{d}^* - \omega_2)(\omega_\mathrm{d}^* - \omega'_+)(\omega_\mathrm{d}^* - \omega'_-) + \frac{\gamma^2}{4}(\omega_\mathrm{d}^* - \omega_1) = 0, \tag{S.78}$$

which has a form identical to that of the Coriolis-coupling system (S.39), except that the eigenfrequencies $\omega_\pm$ [equation (S.27)] are replaced by $\omega'_\pm$ that is given by equation (S.71). The phase-tracked frequencies as functions of the linear-coupling rate g for tuning conditions of $\Delta\omega \approx 0,\ -\gamma,$ and γ are drawn theoretically by the red $\theta_1 = -\pi/2$ contours of the θ_1 responses in Supplementary Fig. **13**a, b, and c, respectively. Here, bifurcation diagrams occur, just like the Coriolis-coupling case in the main text.

Based on the phase-frequency responses of the steady-state (S.76) and (S.77), we obtain the frequency responses of the phase difference for the linear-coupling case,

$$\vartheta = \arctan\frac{\gamma/2}{\omega_\mathrm{d}^* - \omega_2}, \tag{S.79}$$

By substituting (S.79) in the phase-tracked frequency equation (S.78), we obtain the phase difference $\vartheta = \theta_2 - \theta_1$ of the $\theta_1 = -\pi/2$ phase-tracked oscillation of the linear-coupling case, which is given by

$$\gamma^2 \cot\vartheta - 2\Delta\omega\gamma - g^2 \sin\vartheta\cos\vartheta = 0, \tag{S.80}$$

which is different from that of the Coriolis-coupling case [equation (S.50)].

In the degenerate case, the phase-tracked frequency ω_d^* and the phase difference $\vartheta = \theta_2 - \theta_1$ as functions of the linear-coupling strength g, for $g \geq 0$, are shown in Supplementary Fig. **13**d and e, respectively. A spontaneous symmetry breaking at the critical value $g = \gamma$ is revealed as well. However, the polarizations of the phase-tracked oscillations are totally different from those of the Coriolis-coupling case in the main text, due to the difference in ϑ (or θ_2).

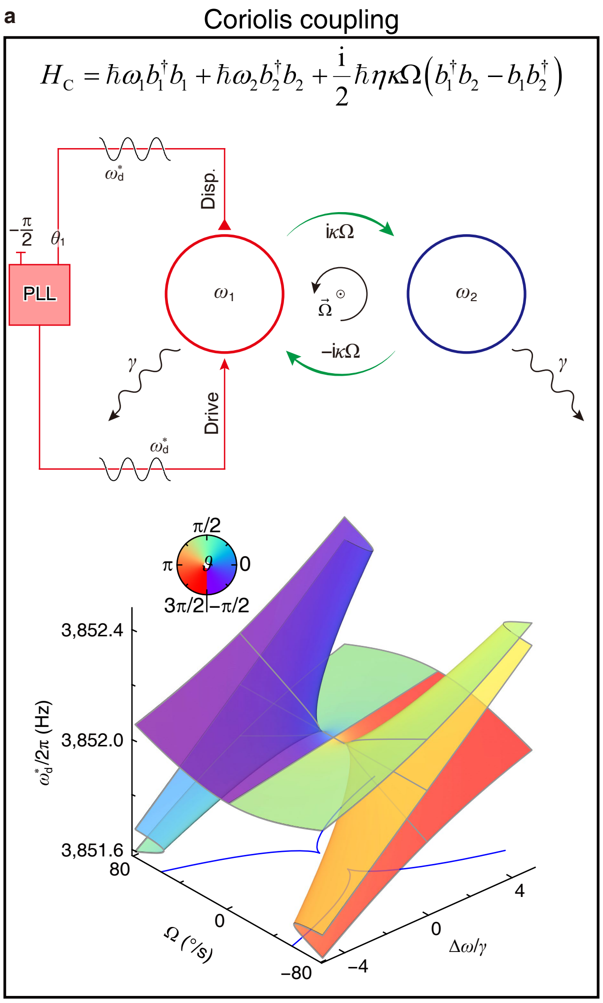

Supplementary Figure 14. **Comparison of the phase-tracked frequencies of the linear-coupling system and Coriolis-coupling system.** (**a**) Hamiltonian of the Coriolis coupling (top). Schematic of the singly driven Coriolis-coupling system with dissipation (middle). Simulated $\theta_1 = -\pi/2$ phase-tracked frequency ω_d^* as a function of angular velocity Ω and natural-frequency difference $\Delta\omega$ (bottom). Colours on the surface represent the relative phase $\vartheta = \theta_2 - \theta_1$ of each point. (**b**) Hamiltonian of the linear coupling (top). Schematic of the singly driven linear-coupling system with dissipation (middle). Simulated $\theta_1 = -\pi/2$ phase-tracked frequency ω_d^* as a function of linear-coupling strength g and natural-frequency difference $\Delta\omega$ (bottom). Mappings of the folded surfaces of both Coriolis coupling and linear coupling to the parameter planes reveal cusp-form singularities. The difference in the ϑ distribution in (a) and (b) indicates that the polarizations of the phase-tracked oscillations of the linear-coupling system are different from those of the Coriolis-coupling system.

Based on equation (S.78), we can draw the phase-tracked frequency ω_d^* of the linear-coupling system in the g-$\Delta\omega$ parameter space, which is compared with that of the Coriolis-coupling case, as shown in Supplementary Fig. **14**. The phase-tracked frequency of the linear-coupling system shows a folded geometry, which is similar to that of the Coriolis-coupled case in Fig. 1d of the main text. The partially folded geometry indicates that cubic cusp singularity can be observed in the linear-coupling system as well.

The mapping of the folded ω_d^*-surface of the linear-coupling system onto the g-$\Delta\omega$ plane shows the loci of the folding (blue curves in Supplementary Fig. **14**b). Cusp-form singularities are revealed, just like the Coriolis-coupling case (blue curves in Supplementary Fig. **14**a or Fig. 1d of the main text).

The cusp singularities at which discontinuous jumps might occur in the linear-coupling system are determined by the discriminant of equation (S.78),

$$16\gamma^2\Delta\omega^4 + (8\gamma^4 + 20g^2\gamma^2 - g^4)\Delta\omega^2 - (g^2-\gamma^2)^3 = 0, \tag{S.81}$$

or explicitly given by

$$\Delta\omega^2 = \frac{g^4}{32\gamma^2} - \frac{5g^2}{8} - \frac{\gamma^2}{4} \pm \sqrt{\frac{g^8}{32^2\gamma^4} + \frac{3g^6}{128\gamma^2} + \frac{3g^4}{16} + \frac{g^2\gamma^2}{2}}. \tag{S.82}$$

It has a very similar form to the Coriolis-coupling expression [equation (S.47)]. Except that the Coriolis-coupling strength $\eta\kappa\Omega$ is replaced by the linear-coupling strength g.

The experimental verification of the phase-tracked singularities in the linear-coupling system remains the next challenging problem, which needs in-situ control of both degeneracy condition and stiffness-coupling strength in an appropriate range [21, 22]. Future studies can also investigate if other complex couplings [23–25] could introduce cubic singularities in the phase-tracked closed-loop oscillations.

SUPPLEMENTARY REFERENCES.

[1] X. Zhou, D. Xiao, X. Wu, Y. Wu, Z. Hou, K. He, and Q. Li, Stiffness-mass decoupled silicon disk resonator for high resolution gyroscopic application with long decay time constant (8.695 s), Applied Physics Letters **109**, 263501 (2016).

[2] Q. Li, D. Xiao, X. Zhou, Y. Xu, M. Zhuo, Z. Hou, K. He, Y. Zhang, and X. Wu, 0.04 degree-per-hour MEMS disk resonator gyroscope with high-quality factor (510 k) and long decaying time constant (74.9 s), Microsystems & Nanoengineering **4**, 32 (2018).

[3] A. D. Challoner, H. G. Howard, and J. Y. Liu, Boeing disc resonator gyroscope, in *IEEE/ION Position, Location and Navigation Symposium-PLANS 2014* (IEEE/ION, Monterey, CA, USA, 2014) pp. 504–514.

[4] D. M. Schwartz, D. Kim, P. Stupar, J. DeNatale, and R. T. M'Closkey, Modal parameter tuning of an axisymmetric resonator via mass perturbation, Journal of Microelectromechanical Systems **24**, 545 (2015).

[5] R. L. Kubena, F. P. Stratton, L. X. Huang, R. J. Joyce, D. J. Kirby, D. T. Chang, and Y. K. Yong, Co-integration of a quartz OCXO and Si MEMS inertial sensors for improved navigational accuracy, in *2016 IEEE International Frequency Control Symposium (IFCS)* (2016) pp. 1–6.

[6] S. H. Nitzan, V. Zega, M. Li, C. H. Ahn, A. Corigliano, T. W. Kenny, and D. A. Horsley, Self-induced parametric amplification arising from nonlinear elastic coupling in a micromechanical resonating disk gyroscope, Scientific Reports **5**, 9036 (2015).

[7] X. Zhou, C. Zhao, D. Xiao, J. Sun, G. Sobreviela, D. D. Gerrard, Y. Chen, I. Flader, T. W. Kenny, X. Wu, and A. A. Seshia, Dynamic modulation of modal coupling in microelectromechanical gyroscopic ring resonators, Nature Communications **10**, 4980 (2019).

[8] A. Schliesser, G. Anetsberger, R. Rivière, O. Arcizet, and T. J. Kippenberg, High-sensitivity monitoring of micromechanical vibration using optical whispering gallery mode resonators, New Journal of Physics **10**, 095015 (2008).

[9] D. Pan, H. Xu, and F. J. G. de Abajo, Rotational Doppler cooling and heating, Science Advances **7**, eabd6705 (2021).

[10] R. Fleury, D. L. Sounas, C. F. Sieck, M. R. Haberman, and A. Alù, Sound isolation and giant linear nonreciprocity in a compact acoustic circulator, Science **343**, 516 (2014).

[11] C. M. Place, *Dynamical Systems: Differential Equations, Maps, and Chaotic Behaviour*, 1st ed. (Routledge, 1992).

[12] T. Gao, E. Estrecho, K. Y. Bliokh, T. C. H. Liew, M. D. Fraser, S. Brodbeck, M. Kamp, C. Schneider, S. Höfling, Y. Yamamoto, F. Nori, Y. S. Kivshar, A. G. Truscott, R. G. Dall, and E. A. Ostrovskaya, Observation of non-Hermitian degeneracies in a chaotic exciton-polariton billiard, Nature **526**, 554 (2015).

[13] J. Doppler, A. A. Mailybaev, J. Böhm, U. Kuhl, A. Girschik, F. Libisch, T. J. Milburn, P. Rabl, N. Moiseyev, and S. Rotter, Dynamically encircling an exceptional point for asymmetric mode switching, Nature **537**, 76 (2016).

[14] H. Xu, D. Mason, L. Jiang, and J. G. E. Harris, Topological energy transfer in an optomechanical system with exceptional points, Nature **537**, 80 (2016).

[15] A. U. Hassan, B. Zhen, M. Soljačić, M. Khajavikhan, and D. N. Christodoulides, Dynamically encircling exceptional points: Exact evolution and polarization state conversion, Physical Review Letters **118**, 093002 (2017).

[16] J. W. Yoon, Y. Choi, C. Hahn, G. Kim, S. H. Song, K.-Y. Yang, J. Y. Lee, Y. Kim, C. S. Lee, J. K. Shin, H.-S. Lee, and P. Berini, Time-asymmetric loop around an exceptional point over the full optical communications

band, Nature **562**, 86 (2018).

[17] X.-L. Zhang, S. Wang, B. Hou, and C. T. Chan, Dynamically encircling exceptional points: In situ control of encircling loops and the role of the starting point, Physical Review X **8**, 021066 (2018).

[18] H. Wang, S. Assawaworrarit, and S. Fan, Dynamics for encircling an exceptional point in a nonlinear non-Hermitian system, Optics Letters **44**, 638 (2019).

[19] Z. Li, C. Li, G. Xu, W. Chen, Z. Xiong, H. Jing, J. S. Ho, and C.-W. Qiu, Synergetic positivity of loss and noise in nonlinear non-Hermitian resonators, Science Advances **9**, eadi0562 (2023).

[20] H. Nasari, G. Lopez-Galmiche, H. E. Lopez-Aviles, A. Schumer, A. U. Hassan, Q. Zhong, S. Rotter, P. LiKamWa, D. N. Christodoulides, and M. Khajavikhan, Observation of chiral state transfer without encircling an exceptional point, Nature **605**, 256 (2022).

[21] P. Thiruvenkatanathan, J. Woodhouse, J. Yan, and A. A. Seshia, Manipulating vibration energy confinement in electrically coupled microelectromechanical resonator arrays, Journal of Microelectromechanical Systems **20**, 157 (2011).

[22] D. K. Agrawal, J. Woodhouse, and A. A. Seshia, Observation of locked phase dynamics and enhanced frequency stability in synchronized micromechanical oscillators, Physical Review Letters **111**, 084101 (2013).

[23] I. Mahboob, K. Nishiguchi, H. Okamoto, and H. Yamaguchi, Phonon-cavity electromechanics, Nature Physics **8**, 387 (2012).

[24] T. Faust, J. Rieger, M. J. Seitner, J. P. Kotthaus, and E. M. Weig, Coherent control of a classical nanomechanical two-level system, Nature Physics **9**, 485 (2013).

[25] S. Gröblacher, K. Hammerer, M. R. Vanner, and M. Aspelmeyer, Observation of strong coupling between a micromechanical resonator and an optical cavity field, Nature **460**, 724 (2009).
